# Unified description of thermal and nonthermal laser-induced ultrafast structural changes in materials

**DOI:** 10.1038/s41598-024-83416-1

**Published:** 2024-12-31

**Authors:** Bernd Bauerhenne, Martin E. Garcia

**Affiliations:** Institute of Physics and Center for Interdisciplinary Nanostructure Science and Technology (CINSaT), University of Kassel, Heinrich-Plett-Strasse 40, 34132 Kassel, Germany

**Keywords:** Silicon, Femtosecond laser excitation, Nonthermal effects, Molecular Dynamics, Computational methods, Phase transitions and critical phenomena, Electronic properties and materials

## Abstract

The ultrafast ionic dynamics in solids induced by intense femtosecond laser excitation are controlled by two fundamentally different yet interrelated phenomena. First, the substantial generation of hot electron-hole pairs by the laser pulse modifies the interatomic bonding strength and characteristics, inducing nonthermal ionic motion. Second, incoherent electron-ion collisions facilitate thermal equilibration between electrons and ions, achieving a uniform temperature on a picosecond timescale. This article presents a unified theoretical description that effectively integrates both processes. Our method is adaptable for use in both ab-initio simulations and extensive molecular dynamics simulations, extending the conventional two-temperature model to incorporate molecular dynamics equations of motion. To demonstrate the efficacy of our approach, we apply it to the laser excitation of silicon thin films. Our simulations closely match experimental observations, accurately reproducing the temporal evolution of the Bragg peaks.

## Introduction

When subjected to an intense femtosecond (fs) laser pulse, a material experiences a dynamic interplay of competing ultrafast processes. Owing to the pronounced interaction of the laser field with electrons and its comparatively minimal interaction with ions, it is generally acknowledged that a transient non-equilibrium state arises following the laser pulse after the thermalization of electrons through electron-electron collisions. This state consists of hot electrons in the conduction band, hot holes in the valence band, and comparatively cold ions^1,2^. The presence of these hot electrons and holes significantly alters the interatomic bonding, resulting in ionic motion that lacks thermal character. This transient state dissipates through incoherent electron-phonon collisions, facilitating energy transfer from electrons to ions, thereby achieving equilibrium between the electronic temperature $T_\text {e}$ and ionic temperature $T_\text {i}$ within a picosecond timescale $\tau _\text {ep}$. The thermal influence of electron-phonon coupling (EPC) on laser-induced structural dynamics has been investigated using the two-temperature-model molecular-dynamics (TTM-MD) simulation approach^3,4^, which utilizes empirical interatomic potentials $V(\textbf{r}_1,\ldots )$ dependent solely on the ionic coordinates $\textbf{r}_1,\ldots$^5,6^. The corresponding equations of motion for both electronic temperature and the ions are formulated as follows:

1 $$\begin{aligned} C_\text {e}(T_\text {e}) \frac{dT_\text {e}}{dt}= & -G_\text {ep}(T_\text {e})\, (T_\text {e} - T_\text {i}) + \frac{dE_{\text {L}_\text {abs}}}{dt}, \end{aligned}$$

2 $$\begin{aligned} M_k\, \frac{d^2\textbf{r}_k}{dt^2}= & -\nabla _{\textbf{r}_k} V(\textbf{r}_1,\ldots ) + \xi \, M_k\, \textbf{v}_k, \end{aligned}$$

where $C_\text {e}(T_\text {e})$ denotes the electronic heat capacity and $G_\text {ep}(T_\text {e})$ represents the electron-phonon coupling coefficient. We want to note that $C_\text {e}$ and $G_\text {ep}$ may also depend on the ionic temperature for strongly coupled electrons and ions^7,8^. The term $dE_{\text {L}_\text {abs}} / dt$ quantifies the rate of energy absorption from the laser, while $-\nabla _{\textbf{r}_k} V(\textbf{r}_1,\ldots )$ represents the conservative force exerted on atom *k*, characterized by mass $M_k$, position $\textbf{r}_k{,}$ and velocity $\textbf{v}_k$. The stokes term $\xi \, M_k\, \textbf{v}_k$ mathematically describes the amplification or damping of the ion velocities due to the electron-phonon coupling, where $\xi$ is given by^4^

3 $$\begin{aligned} \xi = \frac{G_\text {ep}(T_\text {e}-T_\text {i})}{2\, E_\text {kin}}. \end{aligned}$$

$E_\text {kin}$ denotes the kinetic energy of the ions. If the electrons are hotter than the ions, the ions absorb energy from the electrons and become accelerated. If the electrons are colder than the ions, the ions loose energy to the electrons and decelerate. For the sake of simplicity, Eq. (1) and (2) assume homogeneous spatial temperature profiles. It is important to note that the current TTM-MD methodology overlooks a crucial aspect: the impact of hot electrons on interatomic bonding. The generation of hot electrons and holes by the femtosecond laser pulse involves a rearrangement of the occupations of the electronic energy levels. For example, electrons initially in bonding states can be excited into anti-bonding states, altering the bond character. Addressing such rearrangements necessitates a quantum statistical description, which is the foundation for ab-initio molecular dynamics (MD) simulations. In these simulations, a constant volume $\Omega$ simulation cell contains a constant number $N_\text {e}$ of electrons at the temperature $T_\text {e}$, resulting from electron-electron thermalization processes upon laser excitation. For laser excitations producing structural changes, $T_\text {e}$ is in the order of $10^3$-$10^4$ K. The appropriate thermodynamic potential for this situation is the Helmholtz free energy of the electrons, given by

4 $$\begin{aligned} F_\text {e}(T_\text {e}, \Omega , N_\text {e}) = U_\text {e}(S_\text {e}, \Omega , N_\text {e}) - T_\text {e} \, S_\text {e}, \end{aligned}$$

where $U_\text {e}(S_\text {e}, \Omega , N_\text {e})$ is the internal energy, and $S_\text {e}$ is the entropy of the electrons. Both $U_\text {e}$ and $S_\text {e}$ depend on the electronic occupations and the ionic coordinates $\textbf{r}_1, \ldots$. The ab-initio MD simulations describe the motions of the ions classically by

5 $$\begin{aligned} M_k\, \frac{d^2\textbf{r}_k}{dt^2} = -\nabla _{\textbf{r}_k} \Phi (T_\text {e},\textbf{r}_1,\ldots ) \end{aligned}$$

using an effective interatomic potential or potential energy surface (PES) $\Phi (T_\text {e},\textbf{r}_1, \ldots )$ determined by the electrons, which are treated quantum mechanically. For this, a generalized Born-Oppenheimer approximation is used^9^, which yields

6 $$\begin{aligned} \Phi (T_\text {e}, \textbf{r}_1,\ldots ) = U_\text {e}(T_\text {e},\textbf{r}_1,\ldots ) - T_\text {e} \, S_\text {e}(T_\text {e}, \textbf{r}_1,\ldots ). \end{aligned}$$

This means that the PES determining the motion of the ions when the electrons are at temperature $T_\text {e}$ is given by the Helmholz free energy of the electrons. Eq. (6) corresponds to the Mermin free energy [see Eq. (1) in Ref.^10^) used for electronic-temperature dependent density functional theory (DFT)^11,12^. More specifically, the Helmholtz free energy reads in DFT^13^

7 $$\begin{aligned} \Phi (T_\text {e}, \textbf{r}_1,\ldots ) = \sum _m n(\varepsilon _m, T_\text {e}) \, \varepsilon _m + E_\text {XC}\bigl (\rho (\textbf{r})\bigr ) - \int d\textbf{r} \, V_\text {XC}(\textbf{r}) \, \rho (\textbf{r}) - \frac{1}{2} \int d\textbf{r}\, d\textbf{r}' \, \frac{\rho (\textbf{r}) \, \rho (\mathbf {r'})}{|\textbf{r}-\textbf{r}'|} - V_\text {II}(\textbf{r}_1,\ldots ) - T_\text {e} \, S_\text {e}, \end{aligned}$$

where $n(\varepsilon _m, T_\text {e})$ are the electronic occupations of the Kohn-Sham energy levels $\varepsilon _m$. These occupations are given by a Fermi distribution at $T_\text {e}$. $\rho (\textbf{r})$ denotes the electronic charge density

8 $$\begin{aligned} \rho (\textbf{r}) = \sum _m n(\varepsilon _m, T_\text {e}) \, \varphi ^*_m(\textbf{r}) \, \varphi _m(\textbf{r}), \end{aligned}$$

where $\varphi _m(\textbf{r})$ are the Kohn-Sham orbitals. $E_\text {XC}$ represents the exchange and correlation energy, and $V_\text {XC}$ denotes the exchange and correlation potential. $V_\text {II}$ describes the ion-ion repulsion, and the electronic entropy is derived from

9 $$\begin{aligned} S_\text {e} = - k_\text {B} \sum _m \Bigl ( n(\varepsilon _m, T_\text {e}) \, \log \bigl ( n(\varepsilon _m, T_\text {e}) \bigr ) + \bigl ( 1 - n(\varepsilon _m, T_\text {e}) \bigr ) \,\log \bigl ( 1- n(\varepsilon _m, T_\text {e}) \bigr ) \Bigr ) \end{aligned}$$

with $k_\text {B}$ being the Boltzmann constant. The entropy term is crucial here in the canoncial ensemble of the electrons, since otherwise the electronic system is not in thermodynamic equilibrium. Furthermore, it has been shown that the DFT implementation breaks down if the entropy term is ignored^14,15^. Numerous ab-initio MD simulations^16^ have demonstrated that laser excitation significantly alters the PES, resulting in initial non-thermal ionic motion. Such ultrafast nonthermal dynamics facilitate structural transformations that are unattainable in thermodynamic equilibrium, including ultrafast phase transitions^17–20^, thermal phonon squeezing^21,22^, and the generation of coherent phonons^23–25^. Ultrafast x-ray diffraction experiments provide experimental insights into the nonthermal motions^26–29^.

Note, if the electrons are at the ground state ($T_\text {e}=0$), the PES $\Phi (T_\text {e}=0,\textbf{r}_1,\ldots )$ only depends on the ionic coordinates and can eventually be modelled by an analytical interatomic potential $V(\textbf{r}_1,\ldots )$. In fact, $\Phi (T_\text {e}=0,\textbf{r}_1,\ldots )$ is equivalent to $V(\textbf{r}_1,\ldots )$ used in Eq. (2). This indicates a contradiction in the TTM-MD model (1) and (2), since on the one hand it is assumed that the electrons have a finite temperature $T_\text {e}$ but, on the other hand, the forces on the ions related to interatomic bonding do not depend on the electronic temperature. It is important to point out that methods based on the DFT description of the PES are limited to small molecular dynamics cell with almost 1000 atoms and short simulation times not exceeding 10 picoseconds. Moreover, the EPC cannot be included in a clear unified way in DFT.

**Fig. 1:**
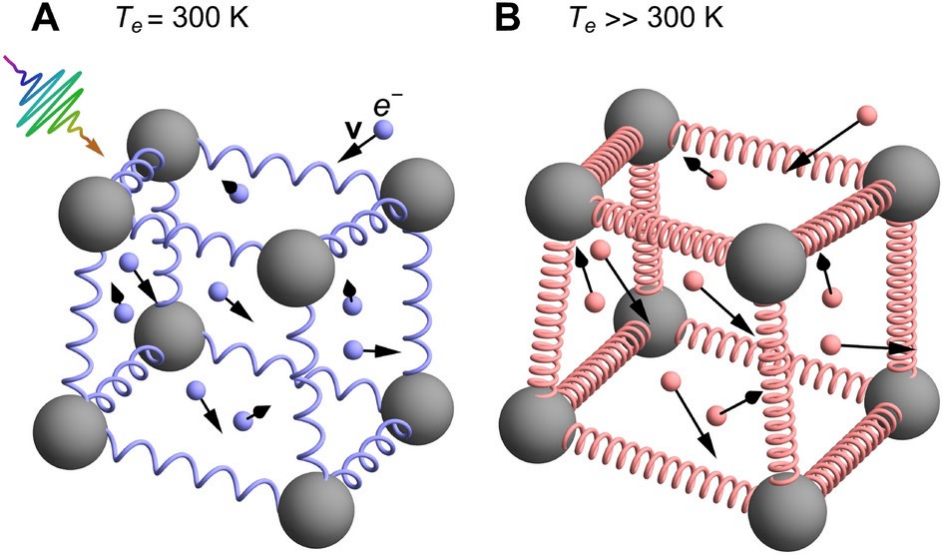
Scheme of the interplay between electron-phonon-coupling (EPC) and laser-induced potential energy surface (PES) changes: Electronic bonds are visualized as springs between the ions that are drawn as grey balls. In addition, the electrons move through the crystal and collide with the ions. These moving electrons are drawn as small balls with black arrows indicating the actual velocity. (**A**) Before the fs-laser excitation the electronic temperature is at 300 K, so that the electrons have a low velocity resulting in infrequent collisions with ions, which vibrate around their equilibrium positions. (**B**) After the femtosecond laser excitation the electrons exhibit a high temperature leading to significant changes of interatomic bonding (red springs) and strong forces acting on the ions. This represents the change of the PES. In addition, the now very fast moving electrons perform strong collisions with the ions (strong EPC).

As one can infer from the previous description (1) - (9), there are two completely different methods for describing the short-time non-thermal dynamics of the ions due to laser-induced bond changes described by the PES and the later structural response initiated by the electron-phonon coupling EPC followed by relaxation of structural stress at times of the order of nanoseconds. The processes dictated by EPC and by the alterations of the PES induced by the laser are in competition (refer to Fig. 1), and thus, they necessitate simultaneous consideration at the same microscopic theoretical level. Furthermore, recent experimental work^30^ emphasizes the importance of integrating both effects to comprehensively understand the mechanism behind laser-induced ultrafast lattice disordering. Despite some endeavors to incorporate both factors^31–33^, a unified first-principles theory has still to be developed. In this paper, we introduce a first-principles derivation of the equations of motion for ions that incorporates the competing influences of EPC and laser-excited PES. We generalize the TTM-MD^4^ approach, enabling it to accommodate the dynamics associated with the excited PES, and adapt the methodology of ab-initio simulations predicated on $T_\text {e}$-dependent PES^11,12,34^, to also consider the impact of EPC. The unified theory developed in this article can considerably improve the atomistic description of laser-processing of materials, covering the whole timescale from the excitation to the final morphology.

## Results

In order to define a proper energy conservation and quantify the energy exchange between electrons and ions, when the effects of the excited PES and the EPC are both present, it is unavoidable to consider ions and electrons as a closed system. In absence of EPC, the ions are already considered as a closed system subject to a PES. However, electrons at a particular temperature $T_\text {e}$ are described in the canonical ensemble, i.e., as an open system in contact with a heat bath. This means that in presence of energy exchange between electrons and ions the electronic system must be formally decoupled from the heat bath and, therefore, be transformed to the microcanonical ensemble. Such a transformation is by no means trivial and is derived in the next subsection.

### Transformation of the electronic system from the canonical to the microcanonical ensemble

We analyze a solid composed of $N_\text {at}$ identical atoms each with mass *M*, excited by a femtosecond laser pulse, resulting in the electrons attaining a uniform temperature $T_\text {e}$. The coordinates $\textbf{r}_i$ of all atoms are collectively represented by the vector $\vec {R} \in \mathbb {R}^{3N_\text {at}}$, and their velocities $\textbf{v}_i$ by the vector $\vec {V} \in \mathbb {R}^{3N_\text {at}}$. The PES of the ions, with the electronic system at temperature $T_\text {e}$, is denoted as $\Phi (T_\text {e}, \vec {R})$. Therefore, the force experienced by atom *i* due to the PES is expressed as $-\nabla _{\textbf{r}_i} \Phi$. The vector $-\nabla _{\vec {R}} \Phi \in \mathbb {R}^{3N_\text {at}}$ encapsulates the forces acting on all atoms. The electronic entropy $S_\text {e}$ and heat capacity $C_\text {e}$ are derived directly from $\Phi$ by

10 $$\begin{aligned} S_\text {e} =&-\frac{\partial \Phi }{\partial T_\text {e}}, \end{aligned}$$

11 $$\begin{aligned} C_\text {e} =&-T_\text {e} \, \frac{\partial ^2 \Phi }{\partial T_\text {e}^2}. \end{aligned}$$

The ions do not exhibit a well defined temperature immediately after laser exictation. Nevertheless, using the kinetic energy $E_\text {kin}$ of the ions and the equipartition theorem, one can assign an average “ionic temperature” $T_\text {i}$ to the ions:

12 $$\begin{aligned} T_\text {i} = \frac{2\, E_\text {kin}}{3 \, N_\text {at} \, k_\text {B}}. \end{aligned}$$

$T_\text {i}$ converges to the actual ionic temperature at longer times. Now we consider the total energy *E* of the whole system as a function of time. If the EPC is not active in the canonical ensemble description, $T_\text {e}$ remains constant and there is no energy exchange between electrons and ions. Then, it holds that

13 $$\begin{aligned} E\bigl .\bigr |_{T_\text {e}} = E_\text {kin} + \Phi \bigl .\bigr |_{T_\text {e}} = \text {const.}, \end{aligned}$$

since the ions form a closed system. Now, if the EPC is active and energy is transferred between electrons and ions, we must transform the electronic system to the microcanonical ensemble in order to be able to treat the whole system of electrons and ions as closed. Based on Eq. (13), we define the term

14 $$\begin{aligned} \tilde{E} := E_\text {kin} + \Phi \overset{(6)}{=} \frac{M}{2} \sum _{j=1}^{N_\text {at}} \textbf{v}_j \cdot \textbf{v}_j + U_\text {e} - T_\text {e}\, S_\text {e}. \end{aligned}$$

We now perform a derivative with respect to the time *t*:

15 $$\begin{aligned} \frac{d\tilde{E}}{dt} =&M \sum _{j=1}^{N_\text {at}} \textbf{v}_j \cdot \frac{d\textbf{v}_j}{dt} + \sum \limits _{j=1}^{N_\text {at}} \left( \nabla _{\textbf{r}_j} U_\text {e} \right) \cdot \frac{d\textbf{r}_j}{dt} + \frac{\partial U_\text {e}}{\partial T_\text {e}} \, \frac{dT_\text {e}}{dt} - T_\text {e} \sum \limits _{j=1}^{N_\text {at}} \left( \nabla _{\textbf{r}_j} S_\text {e} \right) \cdot \frac{d\textbf{r}_j}{dt} -T_\text {e}\,\frac{\partial S_\text {e}}{dT_\text {e}} \, \frac{dT_\text {e}}{dt} - S_\text {e} \, \frac{dT_\text {e}}{dt}. \end{aligned}$$

From this we obtain for the infinitesimal change $d\tilde{E}$ for an infinitesimal time change *dt*

16 $$\begin{aligned} d\tilde{E} =&M \sum _{j=1}^{N_\text {at}} \textbf{v}_j \cdot \frac{d\textbf{v}_j}{dt} \, dt + \sum \limits _{j=1}^{N_\text {at}} \left( \nabla _{\textbf{r}_j}\Phi \right) \cdot \frac{d\textbf{r}_j}{dt} \, dt + \frac{\partial U_\text {e}}{\partial T_\text {e}} \, \frac{dT_\text {e}}{dt} \, dt - \left( T_\text {e}\,\frac{\partial S_\text {e}}{\partial T_\text {e}} + S_\text {e} \right) \, \frac{dT_\text {e}}{dt} \, dt. \end{aligned}$$

The last two terms transform dynamically the electrons from the microcanonical to the canonical ensemble, so that we have to remove these terms in order to get the infinitesimal energy change *dE*:

17 $$\begin{aligned} dE = d\tilde{E} + \left( T_\text {e}\,\frac{\partial S_\text {e}}{\partial T_\text {e}} + S_\text {e} \right) \, \frac{dT_\text {e}}{dt} \, dt. \end{aligned}$$

For obtaining the energy $E(t_1)$ at time $t_1$, we integrate the above expression starting from a reference time $t_0$:

18 $$\begin{aligned} E(t_1) = \tilde{E}(t_1) -\tilde{E}(t_0) + \int \limits _{t_0}^{t_1} dt \, \left( T_\text {e}\,\frac{\partial S_\text {e}}{\partial T_\text {e}} + S_\text {e} \right) \, \frac{dT_\text {e}}{dt}. \end{aligned}$$

The energy is only defined up to a constant, so that we can set $\tilde{E}(t_0)=0$. If we insert Eq. (10) for $S_\text {e}$ and Eq. (14) for $\tilde{E}(t_1)$, we obtain finally

19 $$\begin{aligned} \boxed {E(t_1) = E_\text {kin}(t_1)+ \Phi (t_1) - \int \limits _{t_0}^{t_1} dt \left( T_\text {e}\, \frac{\partial ^2 \Phi }{\partial T_\text {e}^2} + \frac{\partial \Phi }{\partial T_\text {e}} \right) \frac{dT_\text {e}}{dt}.} \end{aligned}$$

Equation (19) represents the central equation of this article, enabling the simultaneous consideration of laser-induced modifications to the PES and the EPC within the same ab initio theoretical framework. Notice, that only using Eq. (19) one can ensure the energy conservation in the whole system consisting of electrons and ions. Previous formulation^32^ used $E = E_\text {kin}+ U_\text {e}$ for the total energy. However, this has been shown to lead to inconsistencies and to wrong expression for interatomic forces^14,15^. Now we are able to formulate the energy conservation for moving ions and changing $T_\text {e}$. Now we are positioned to articulate the energy conservation for moving ions and the varying temperature $T_\text {e}$. Given that the entire system operates within the microcanonical ensemble, we are able to account for the effect of the total energy absorbed from the laser up until time $t_1$, denoted as $E_{\text {L}_\text {abs}}(t_1),$ as:

20 $$\begin{aligned} E(t_1) = E_{\text {L}_\text {abs}}(t_1) + \text {const.} \end{aligned}$$

To derive the equations of motion, we compute the time derivative of the energy *E* as presented in Eq. (19).

21 $$\begin{aligned} \frac{dE}{dt}= & M \sum _{j=1}^{N_\text {at}} \textbf{v}_j \cdot \frac{d\textbf{v}_j}{dt} + \sum _{j=1}^{N_\text {at}} \textbf{v}_j \cdot \nabla _{\textbf{r}_j}\Phi - T_\text {e}\, \frac{\partial ^2 \Phi }{\partial T_\text {e}^2} \, \frac{dT_\text {e}}{dt} \nonumber \\\overset{(11)}{=} & M\, \vec {V} \cdot \frac{d\vec {V}}{dt} +\vec {V} \cdot \nabla _{\vec {R}} \Phi + C_\text {e} \, \frac{dT_\text {e}}{dt}. \end{aligned}$$

The third term corresponds to the time derivative of the internal energy of the electrons

22 $$\begin{aligned} \frac{dE_\text {e}}{dt} = C_\text {e} \, \frac{dT_\text {e}}{dt}. \end{aligned}$$

Given that we omit local electronic heat flow by utilizing a global $T_\text {i}$ and $T_\text {e}$, we consider only two processes that affect the internal energy $E_\text {e}$ of the electrons: The energy exchange between electrons and ions facilitated by EPC. We denote the cumulative energy exchanged between the electrons and ions up to time $t_1$ as $E_\text {ep}(t_1)$. The coupling $G_{\text {ep}}$ is, in general, a function of $T_\text {e}$, $T_\text {i}$ and the ionic coordinates $\textbf{r}_1,\ldots$ and indicates the magnitude of the energy flow from the phonons to the electrons depending on the temperature difference $T_\text {e}-T_\text {i}$ and is an external parameter for us. Additionally, the electrons have the capability to absorb energy directly from a laser field. Consequently, the time derivative of the internal energy $E_\text {e}$ of the electrons can be expressed as follows:

23 $$\begin{aligned} \underbrace{C_\text {e} \, \frac{dT_\text {e}}{dt}}_{\overset{(22)}{=}\frac{dE_\text {e}}{dt}} = - G_\text {ep} \left( T_\text {e} - T_\text {i}\right) + \frac{dE_{\text {L}_\text {abs}}}{dt}. \end{aligned}$$

This differential equation governs the time variation of the electronic temperature $T_\text {e}$, which is similarly employed in the two-temperature model (TTM)^35^ and TTM-MD approaches^4^, under the assumption of uniform global ionic and electronic temperatures. The equations of motion for the ions are derived from the time derivative of the energy conservation expressed in Eq. (20):

24 $$\begin{aligned} \frac{dE}{dt} = \frac{dE_{\text {L}_\text {abs}}}{dt}. \end{aligned}$$

Inserting Eq. (21) and using Eq. (23) for $C_\text {e} \, \frac{dT_\text {e}}{dt}$ we find

$$\begin{aligned} M\, \vec {V} \cdot \frac{d\vec {V}}{dt} + \vec {V} \cdot \nabla _{\vec {R}} \Phi - \underbrace{\frac{M\, \vec {V} \cdot \vec {V} }{2\, E_\text {kin}}}_{=1} \, G_\text {ep} \left( T_\text {e} - T_\text {i}\right) =&0, \nonumber \\ \Leftrightarrow \qquad \vec {V} \cdot \left( M\, \frac{d\vec {V}}{dt} + \nabla _{\vec {R}} \Phi - \frac{G_\text {ep} \left( T_\text {e} - T_\text {i}\right) }{2\, E_\text {kin}} \, M\, \vec {V} \right) =&0. \end{aligned}$$

The above equation must be valid for arbitrary velocities $\vec {V}$. Therefore, it must hold that

25 $$\begin{aligned} M\, \frac{d\vec {V}}{dt} = - \nabla _{\vec {R}} \Phi + \frac{G_\text {ep} \left( T_\text {e} - T_\text {i}\right) }{2\, E_\text {kin}}\, M \, \vec {V}. \end{aligned}$$

The first term on the right-hand side represents the force derived from the PES at temperature $T_\text {e}$, and the second term corresponds to the force attributable to EPC. The collective set of equations, labeled (19), (23), and (25), encapsulates the unified theoretical framework developed in this article. We now proceed to analyze two significant limiting cases:

1. When $T_\text {e}$ remains constant, Eq. (19) simplifies to $E(t_1) = E_\text {kin}(t_1) + \Phi (t_1)$, as the integral term becomes zero due to the condition $dT_\text {e} / dt = 0$. Therefore, under the conditions of constant $T_\text {e}$ and no additional energy absorption from the laser, such that $E_{\text {L}_\text {abs}} \equiv 0$, Eq.(20) transitions to Eq. (13). This form is frequently employed in $T_\text {e}$-dependent DFT MD simulations that are conducted at a constant $T_\text {e}$^12,22,34^.
2. If the changes of the PES due to the laser excitation are ignored, i.e., the electrons are considered to be in their ground state, it holds that $\Phi \equiv \Phi (T_\text {e}=0)$ and $d\Phi / dT_\text {e} =0$. Since $\Phi (T_\text {e}=0,\textbf{r}_1,\ldots ) = U_\text {e}(\textbf{r}_1),$ Eqs. (23) and (25) turn into the commonly used TTM-MD Eqs. (1), (2), if $C_\text {e}$ is used as an external parameter instead of being directly calculated from $\Phi$ via Eq. (11). This implies that conventional TTM-MD approaches, which rely on empirical interatomic potentials solely based on atomic coordinates, implicitly assume that the electrons are perpetually in their ground state.

Both limits show the power of the developed theory, which, on the one side, generalizes the TTM-MD equations including PES effects and, on the other side, contains both the usual TTM-MD model and the ab-initio approaches considering only the PES changes as limiting cases.

### Simulations and comparison with experiments

In order to confirm the validity our method, we performed MD simulations applying the theory derived in this paper and using the $T_\text {e}$-dependent interatomic potential $\Phi ^{(\text {Si})}(T_\text {e})$ for Si derived in^36^ from DFT in the LDA approximation and compared directly with experimental results by Harb *et al.*^28,29^ on free-standing thin Si films. We used the POLYPOT1_MD_MPI 2.0 code^37^. We used for the electron-phonon coupling the constant $G_{\text {ep}} = 1.8 \times 10^{17}\frac{\text {W}}{\text {K }\text { m}^3}$ for Si derived from ab initio in^38^. We used for Si, although it is a semiconductor, a common chemical potential for electrons and holes. This is possible, since silicon (Si) becomes metallic after the laser excitation due to the atomic disorder^39^. To determine the performance of the theory developed here with previous approaches, we performed MD simulations in three different scenarios:

1. We performed the MD simulations by integrating the Eqs. (23) and (25) and using the expression (19) for the total energy. In this way, we consider the effects of the excited PES and the EPC on the same theory level as mentioned through the paper.
2. We only consider the effect of the excited PES. This is achieved by setting $G_\text {ep}=0$ in Eqs. (23) and (25). In this way, we mimic the ab-initio simulations based on $T_\text {e}$-dependent DFT.
3. We only consider the effect of the electron-phonon coupling (EPC). In this scenario, within Eqs. (23) and (25), we use for $\Phi$ the expression $\Phi ^{(\text {Si})}(T_\text {e}=0) + E_\text {e}(T_\text {e})$. This indicates that the PES is consistently evaluated at $T_\text {e}=0$, signifying that the bonding is described by electrons in their ground state. The additional term, $E_\text {e}(T_\text {e})$, represents the electronic energy as a function of $T_\text {e}$, as derived from DFT for the ideal crystal structure (refer to methods). This inclusion ensures the accurate calculation of the electronic heat capacity $C_\text {e}(T_\text {e})$ from Eq. (11). In this way, we reproduce the pure TTM-MD method based on interatomic potentials only depending on the atomic coordinates.

**Fig. 2:**
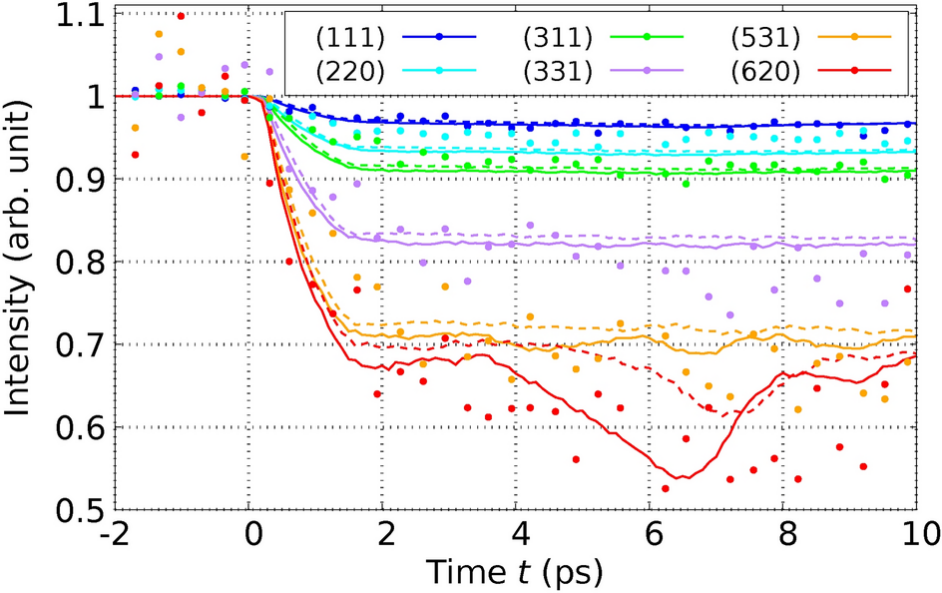
Relative intensities of various Bragg peaks of a 50-nm thick Si film after laser-excitation with a fluence below the damage threshold are shown as a function of time. The data points in the graph correspond to experimental results, while the lines depict values calculated from our MD simulations. Solid lines represent calculations that take into account both the excited PES and EPC, whereas dashed lines pertain to simulations considering only the EPC. The experimental values are extracted from Fig. 4 of Reference^28^.

**Fig. 3:**
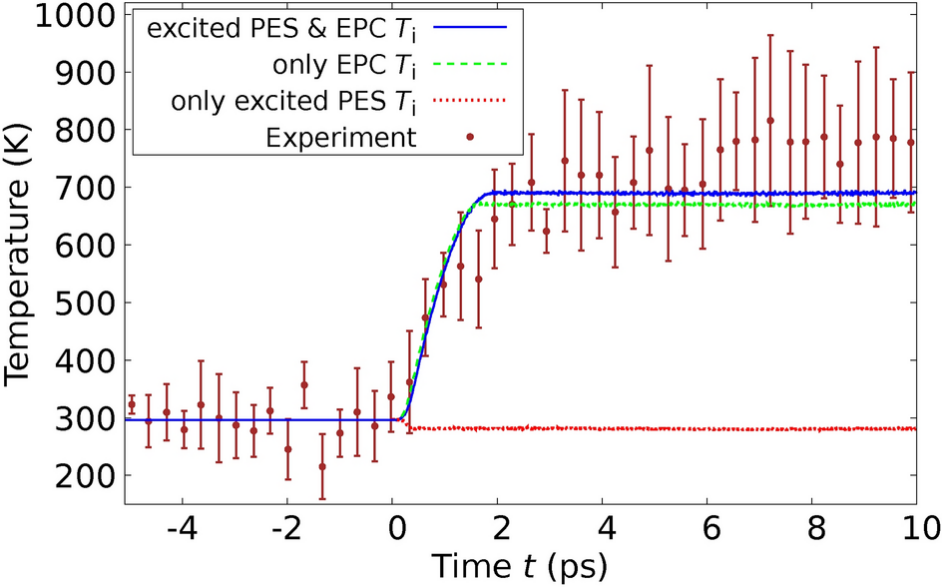
Ionic temperature $T_\text {i}$ of the 50-nm thick Si film after laser-excitation with a fluence below the damage threshold is shown as a function of time. The points with error bars correspond to the experiment and were determined using the Debye Waller theory. Lines refer to our calculated values. The experimental values are taken from Fig. 5 of Ref.^28^.

In an initial experiment, Harb *et al.* utilized a fs laser to excite a Si film with a thickness of $d_\text {film} = 50 \, \text {nm}$ at a fluence of $I_{\text {L}_\text {tot}} = 5.6 \, \text {mJ} / \text {cm}^2{,}$ which remains below the threshold for damage^28^. This fluence equates to an absorbed energy per atom of $E_{L_\text {abs}} / N_\text {at} = 0.1 \, \text {eV} / \text {atom}$, calculated using Eq. (59) (refer to Methods). Harb *et al.* employed ultrafast electron diffraction to observe the time-dependent intensities of various Bragg peaks. To conduct a direct comparison with this experiment, we configured a simulation cell encompassing $11 \times 11 \times 93$ conventional cells, which incorporated a Si film 50 nm in thickness, containing a total of $N_\text {at} = 90024$ atoms. In Figure 2, we juxtapose the experimentally measured relative intensities with those generated from our simulations for the six Bragg peaks analyzed by Harb *et al.* The details the calcuation of the Bragg peaks from the MD simulations are given in Methods. The relative Bragg peak intensities obtained from the MD simulations considering both effects – excited PES & EPC – yields good agreement with the experiments. Notice, however, that simulations using the constraint (3), i.e., considering only the EPC effect are almost identical with the full calculation. From this fact we conclude that in those experiments at low fluences the ionic motions are clearly dominated by the EPC. Simulations using the constraint (2), i.e., considering only the effect of the excited PES, yield a featureless behaviour of the Bragg peak intensities as function of time. In addition to analyzing the Bragg peaks, Harb *et al.* also calculated the time-dependent ionic temperature $T_\text {i}$ of the Si film. This was achieved by interpreting the temporal changes in Bragg peak intensities through the application of Debye-Waller theory. We calculated the ionic temperature directly from the simulations using Eq. (12). Our results reproduce the measured $T_\text {i}$, as one can observe in Fig. 3. As expected from the previous Fig. 2, the full simulation and the simulation with constraint (3) yield almost the same curve for the ionic temperature. Interestingly, simulations using the constraint (2) yields a constant ionic temperature and completely disagree with the experiments.

**Fig. 4:**
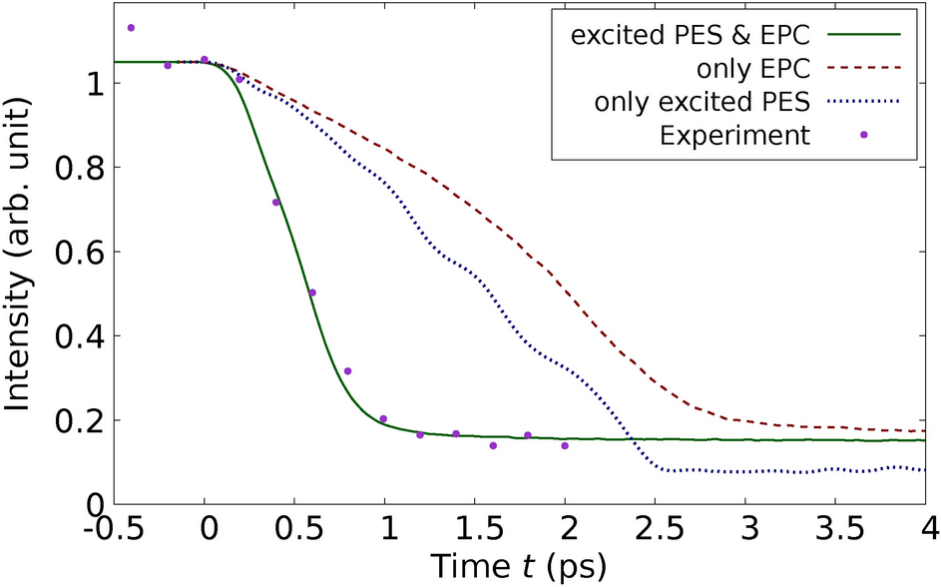
Relative intensity of the (220) Bragg peak of the 30-nm thick Si film after laser-excitation with a fluence above the damage threshold is shown as a function of time. Points refer to the experiment and lines correspond to our calculations. The solid line represents the full calculation including the influence of the excited PES & EPC. Notice the excellent agreement with experiment. The dotted line refers to the calculated values using the constrained simulations only including excited PES. The dashed line corresponds to the calculated values considering only the effect of the EPC. The experimental values are taken from Fig. 3 (c) of Ref.^29^.

In a subsequent experiment, Harb *et al.* applied a fs laser pulse to excite a Si film with a thickness of $d_\text {film} = 30 \, \text {nm}.$ The laser fluence used, $I_{\text {L}_\text {abs}} = 65 \, \text {mJ} / \text {cm}^2$, was above the damage threshold for the material, as documented in their study^29^. This fluence corresponds to $E_{L_\text {abs}} / N_\text {at} = 1.2\, \textrm{eV} / \textrm{atom}$ using Eq. (59). For the simulations corresponding to the setup by Harb *et al.*, a simulation cell was configured consisting of $11 \times 11 \times 56$ conventional cells. This assembly contains a Si film with a thickness of $30 \, \text {nm}$ and is composed of $N_\text {at} = 54208$ atoms. In Figure 4, we present the relative intensity of the (220) Bragg peak. This figure includes both the results obtained from our computational simulations and those from the experiment, facilitating a direct comparison.

In this case, we also applied the three types of MD simulations described above. It is important to note that when MD simulations account only for the effect of the excited PES, as is common in standard DFT approaches, or solely the effect of EPC, as typical in traditional TTM-MD simulations using empirical potentials, there is a significant deviation from the experimental results. Specifically, when only the effects of the excited PES are considered, the simulations display oscillations in the Bragg peak intensity and exhibit a much slower decay than what is observed experimentally. This discrepancy highlights the necessity of incorporating both PES and EPC effects to accurately model the dynamics observed in experimental conditions. The oscillations have their origin in the laser-induced movement of the crystal planes against each other. Such an oscillation of the Bragg peak intensity is not observed in the experiment. When only the effects of EPC are considered, the immediate melting of the crystal planes is observed, and no oscillations are evident in the simulation results. Additionally, the decay of Bragg peak intensities in this scenario is both quantitatively and qualitatively different from experimental observations, presenting an even slower decline than when only the influence of the excited PES is considered. In contrast, simulations that incorporate both the excited PES and EPC, as dictated by the comprehensive theory presented in this paper, achieve remarkable agreement with the experimental results. Importantly, these results are obtained without the use of adjusting parameters, underscoring the robustness of the theoretical approach. As demonstrated in Figure 4, at high fluences, it is crucial to account for both competing contributions-the modifications in the PES and the effects of EPC. This comprehensive modeling approach is essential for accurately replicating the experimental outcomes observed under such conditions. Our findings of the dominance of EPC at low fluences and the increasing importance of nonthermal effects at high fluences for Si agree also with the results of^40,41^.

## Discussion

While the theory outlined in this article successfully integrates thermal (incoherent electron-phonon heating) and non-thermal (bond changes) effects in solids following laser excitation, there are opportunities for further refinements, particularly through the incorporation of electron dynamics during laser excitation and the subsequent electron thermalization. To date, such an extensive theoretical framework has not been established.

Time-Dependent Density Functional Theory (TDDFT), as referenced by Runge and Gross^42^ and further explored in the works of Wijewardane and Ullrich^43^, and Krishna *et al.*^44^, is proficient in accurately simulating the interaction between the laser pulse and electrons. However, TDDFT encounters significant challenges in describing dephasing effects and the process of electron thermalization. This limitation confines its applicability primarily to the time frame while the laser pulse is active. Recently, a modified real time TDDFT was developed to include, in some way, dephasing and a detailed balance on the electronic occupations^45^. However, such calculations are restricted to a very small number of atoms. In contrast, methods grounded in Boltzmann collision integrals^46,47^ successfully model electron thermalization but operate normally with fixed ionic positions and were recently extended to moving ions^48^. The accuracy of the method detailed in this article is expected to become highly reliable once the electronic temperature, $T_\text {e}$, stabilizes. This stabilization typically occurs within a timeframe ranging from 50 to 100 femtoseconds.

In summary, this article presents a unified theory that effectively describes the structural changes induced by laser excitation in solids, integrating both bond modifications and electron-phonon coupling at a microscopic level. Our approach encompasses and extends the traditional frameworks of TTM-MD and ab-initio DFT simulations, treating them as special cases. When applied to Si, our methodology demonstrated remarkable concordance with experimental findings, validating its accuracy in capturing the complex dynamics associated with laser-induced structural transformations. This success underscores the potential of our unified approach to provide a comprehensive and accurate tool for studying and predicting laser-induced phenomena in various materials.

## Methods

Now we explain in detail, how we performed our MD simulations. At first, we show the generalized formulation of our theory for separate phonon temperatures, which we did not present in the main text for the sake of simplicity. The algorithm for the integration of the equations of motion are implemented considering this generalized formulation.

### Generalization of the atomic equations of motion considering separate phonon temperatures

Indeed, it is feasible to assign specific ionic temperatures to distinct sets of phonon modes, as outlined by Waldecker et al.^49^. The process begins with the diagonalization of the dynamical matrix, from which one can derive the polarization vectors $\vec {e}^{\;(1)},\ldots , \vec {e}^{\;(3\,N_\text {at})}$ for all phonon modes. These vectors are orthonormal and together form a complete basis set of the vector space $\mathbb {R}^{3\, N_\text {at}}$. The phonon modes are then categorized into $N_\mathscr {M}$ separate subsets, denoted as $\mathscr {M}_k$. The number of phonon modes within each subset $\mathscr {M}_k$ is represented by $|\mathscr {M}_k|$. For each subset $\mathscr {M}_k$, one can define the corresponding projection operator $\textbf{P}_{\mathscr {M}_k}$ which is expressed as a matrix in $\mathbb {R}^{3\,N_\text {at} \times 3\,N_\text {at}}$. The projection operator is constructed as follows:

26 $$\begin{aligned} \textbf{P}_{\mathscr {M}_k} = \sum _{j \in \mathscr {M}_k} \vec {e}^{\;(j)} \cdot \left( \vec {e}^{\;(j)}\right) . \end{aligned}$$

This operator projects the atomic velocities $\vec {V}$ onto the directions of the phonon modes of set $\mathscr {M}_k$, obeys $\textbf{P}_{\mathscr {M}_k}^t = \textbf{P}_{\mathscr {M}_k} = \textbf{P}_{\mathscr {M}_k}^2$, and is used to define an individual ionic temperature for a given set $\mathscr {M}_k$ of phonon modes:

27 $$\begin{aligned} T_{\text {i}_{\mathscr {M}_k}} = \frac{2\, E_{\text {kin}_{\mathscr {M}_k}}}{|\mathscr {M}_k| \, k_\text {B}} = \frac{M\, \vec {V} \cdot \textbf{P}_{\mathscr {M}_k} \cdot \vec {V}}{|\mathscr {M}_k| \, k_\text {B}}. \end{aligned}$$

Here $k_\text {B}$ denotes the Boltzmann constant and $E_{\text {kin}_{\mathscr {M}_k}}$ the kinetic energy of the phonon modes of set $\mathscr {M}_k$. Now, the time derivative of the internal energy of the electrons is given by

28 $$\begin{aligned} \underbrace{C_\text {e} \, \frac{dT_\text {e}}{dt}}_{\overset{(22)}{=}\frac{dE_\text {e}}{dt}} = -\sum _{k=1}^{N_\mathscr {M}} |\mathscr {M}_k| \, G_{\text {ep}_{\mathscr {M}_k}} \left( T_\text {e} - T_{\text {i}_{\mathscr {M}_k}}\right) + \frac{dE_{\text {L}_\text {abs}}}{dt}. \end{aligned}$$

The equations of motions for the ions is calculated from the time derivative of Eq. (20):

29 $$\begin{aligned} \frac{dE}{dt} = \frac{dE_{\text {L}_\text {abs}}}{dt}. \end{aligned}$$

Inserting Eq. (21) and using Eq. (23) for $C_\text {e} \, \frac{dT_\text {e}}{dt}$ we find

$$\begin{aligned} M\, \vec {V} \cdot \frac{d\vec {V}}{dt} + \vec {V} \cdot \nabla _{\vec {R}} \Phi - \sum _{k=1}^{N_\mathscr {M}} \underbrace{\frac{M\, \vec {V} \cdot \textbf{P}_{\mathscr {M}_k} \cdot \vec {V} }{2\, E_{\text {kin}_{\mathscr {M}_k}}}}_{=1} \, |\mathscr {M}_k| \, G_{\text {ep}_{\mathscr {M}_k}} \left( T_\text {e} - T_{\text {i}_{\mathscr {M}_k}}\right) = 0, \end{aligned}$$

The above equation must be valid for arbitrary velocities $\vec {V}$. Therefore, it must hold that

30 $$\begin{aligned} M\, \frac{d\vec {V}}{dt} = - \nabla _{\vec {R}} \Phi + \sum _{k=1}^{N_\mathscr {M}} \frac{|\mathscr {M}_k| \, G_{\text {ep}_{\mathscr {M}_k}} \left( T_\text {e} - T_{\text {i}_{\mathscr {M}_k}}\right) }{2\, E_{\text {kin}_{\mathscr {M}_k}}}\, M \, \textbf{P}_{\mathscr {M}_k} \cdot \vec {V}. \end{aligned}$$

The first term on the right-hand side represents the force arising from the PES at the electronic temperature $T_\text {e}$, while the second term corresponds to the force resulting from EPC.

### Implementation in the velocity verlet algorithm

We denote by $\vec {F}$ the vector containing all interatomic forces related to the potential energy surface

31 $$\begin{aligned} \vec {{F}} =&- \nabla _{\vec {R}} \, \Phi \end{aligned}$$

and by $\vec {{F}}_\text {tot}$ the vector containing the total interatomic forces

32 $$\begin{aligned} \vec {{F}}_\text {tot} =&\vec {{F}} + \sum _{k=1}^{N_\mathscr {M}} \, \xi _{\mathscr {M}_k} \, M \, \textbf{P}_{\mathscr {M}_k} \cdot \vec {{V}}, \end{aligned}$$

33 $$\begin{aligned} \xi _{\mathscr {M}_k} =&\frac{|\mathscr {M}_k| \, G_{\text {ep}_{\mathscr {M}_k}} \left( T_\text {e} - T_{\text {i}_{\mathscr {M}_k}}\right) }{2\, E_{\text {kin}_{\mathscr {M}_k}}}. \end{aligned}$$

In any MD simulation, the process begins by establishing the initial conditions, which include the electronic temperature at the initial time $T_\text {e}(t_0)$, the initial positions of the atoms $\vec {R}(t_0)$, and their initial velocities $\vec {V}(t_0)$. A positive time increment $\Delta t$ is chosen, and the simulation evaluates the system at discrete times $t_\ell = t_0 + \ell \Delta t$, where $\ell$ belongs to the set of natural numbers $\mathbb {N}$. At these times, the simulation aims to calculate the electronic temperature $T_\text {e}(t_\ell )$, the positions $\vec {R}(t_\ell )$, and the velocities $\vec {V}(t_\ell )$ of the atoms. To achieve these calculations, it is necessary to numerically integrate the coupled differential equations governing the motions of the ions

$$\begin{aligned} M\, \frac{d\vec {{V}}}{dt} = \vec {{F}}_\text {tot} \end{aligned}$$

and the differential equation for $T_\text {e}$

$$\begin{aligned} C_\text {e}\, \frac{dT_\text {e}}{dt} = \frac{dE_\text {e}}{dt}. \end{aligned}$$

The Velocity Verlet Algorithm^50^ is a popular choice for integrating the equations of motion in molecular dynamics simulations due to its simplicity and numerical stability. The algorithm updates positions, velocities, and forces of the atoms at each timestep based on their values from the previous timestep. The process involves the following steps:

34 $$\begin{aligned} \vec {{R}}(t_{\ell +1}) =&\vec {{R}}(t_\ell ) + \Delta t \ \vec {{V}}(t_\ell ) + \frac{\Delta t^2}{2\,M} \, \vec {{F}}_\text {tot}(t_\ell ), \end{aligned}$$

35 $$\begin{aligned} \vec {{V}}(t_{\ell +1}) =&\vec {{V}}(t_\ell ) + \frac{\Delta t}{2\, M} \, \Bigl (\vec {{F}}_\text {tot}(t_\ell ) + \vec {{F}}_\text {tot}(t_{\ell +1}) \Bigr ). \end{aligned}$$

Utilizing the initial position vector $\vec {R}(t_0)$, we are equipped to compute $\vec {F}(t_0)$, $\Phi (t_0)$, $S_\text {e}(t_0)$, and $C_\text {e}(t_0)$. Furthermore, the phonon mode projection operators $P_{\mathscr {M}_k}$, which are presumed to be time-invariant, are acknowledged. These projection operators $P_{\mathscr {M}_k}$, in conjunction with $\vec {V}(t_0)$, facilitate the calculation of kinetic energies $E_{\text {kin}_{\mathscr {M}_k}}(t_0)$, temperatures $T_{\text {i}_{\mathscr {M}_k}}(t_0)$, and the parameters $\xi _{\mathscr {M}_k}(t_0)$ for various phonon mode groups $\mathscr {M}_k$. As a result, the total force vector $\vec {F}_\text {tot}(t_0)$ at the initial time $t_0$ can be immediately derived from the initial conditions, as formulated in Eq. (32). From the variables $\vec {R}(t_0)$, $\vec {V}(t_0)$, and $\vec {F}_\text {tot}(t_0)$, we proceed to compute the position vector $\vec {R}(t_1)$ using Eq.(34).

Nevertheless, direct computation of $\vec {V}(t_1)$ from Eq. (35) is not possible, since it requires $\vec {F}_\text {tot}(t_1)$, which in turn can only be determined from $\vec {V}(t_1)$ via Eq. (32). In scenarios where electron-phonon coupling is disregarded, $\vec {F}_\text {tot}(t_1)$ can be directly computed from $\vec {R}(t_1)$ utilizing $\Phi$, given that $\vec {F}_\text {tot}(t_1)$ simplifies to $\vec {F}(t_1)$. Subsequently, $\vec {V}(t_1)$ can be calculated straightforwardly using Eq. (35).

In scenarios where electron-phonon coupling must be accounted for, we must adjust the computational procedure as follows: We start from a time step $t_\ell \ge t_0$ where all known quantities are established and aim to compute all relevant quantities at the subsequent time step $t_{\ell +1}$. Initially, $\vec {R}(t_{\ell +1})$ is determined using Eq. (34). To accurately calculate $T_\text {e}(t_{\ell +1})$, additional definitions and steps are required. We define

36 $$\begin{aligned} \Delta E_{\text {L}_\text {abs}}(t_\ell ) = \int \limits _{t_\ell }^{t_{\ell +1}} dt\, \frac{dE_{\text {L}_\text {abs}}(t)}{dt} = E_{\text {L}_\text {abs}}(t_{\ell +1}) - E_{\text {L}_\text {abs}}(t_\ell ) \end{aligned}$$

which represents the energy absorbed by electrons from the laser during the time step $t_\ell$. Additionally, $\Delta E_\text {ep}(t_\ell )$ is defined as the total energy transferred to the electrons from the ions as a result of electron-phonon coupling at the same time step $t_\ell$. The numerical computation of $\Delta E_\text {ep}(t_\ell )$ is conducted by

37 $$\begin{aligned} \Delta E_\text {ep}(t_\ell ) = -\sum _{k=1}^{N_\mathscr {M}} |\mathscr {M}_k| \, G_{\text {ep}_{\mathscr {M}_k}}(t_\ell ) \left( T_\text {e}(t_\ell ) - T_{\text {i}_{\mathscr {M}_k}}(t_\ell )\right) \Delta t. \end{aligned}$$

From the total change of the electronic energy at time step $t_\ell$

38 $$\begin{aligned} \Delta E_\text {e}(t_\ell ) = \Delta E_\text {ep}(t_\ell ) + \Delta E_{\text {L}_\text {abs}}(t_\ell ), \end{aligned}$$

we can calculate numerically the related change of $T_\text {e}$ for $C_\text {e}(t_\ell )>0$ by

39 $$\begin{aligned} \Delta T_\text {e}(t_\ell ) = \frac{\Delta E_\text {e}(t_\ell )}{C_\text {e}(t_\ell )} \overset{(38)}{=} \frac{\Delta E_\text {ep}(t_\ell ) + \Delta E_{\text {L}_\text {abs}}(t_\ell )}{C_\text {e}(t_\ell )}. \end{aligned}$$

From $\Delta T_\text {e}(t_\ell )$, we obtain $T_\text {e}(t_{\ell +1})$ just by

40 $$\begin{aligned} T_\text {e}(t_{\ell +1}) = T_\text {e}(t_\ell ) + \Delta T_\text {e}(t_\ell ). \end{aligned}$$

In cases where $C_\text {e}(t_\ell )=0$, we formally consider the ions to be stationary and attribute any change in internal energy, provoked by variations in the electron temperature $T_\text {e}$, exclusively to the electron subsystem. This assumption simplifies the energy transfer dynamics by isolating the electron behavior from the ionic lattice

41 $$\begin{aligned} \Delta E_\text {e}(t_\ell ) =&\Delta E(t_\ell ) \nonumber \\ =&\Phi \left( T_\text {e}(t_{\ell +1}), \vec {{R}}(t_\ell ) \right) - T_\text {e}(t_{\ell +1}) \, \frac{d\Phi \left( T_\text {e}(t_{\ell +1}), \vec {{R}}(t_\ell ) \right) }{dT_\text {e}} \nonumber \\&- \Phi \Bigl (T_\text {e}(t_\ell ), \vec {{R}}(t_\ell ) \Bigr ) + T_\text {e}(t_\ell ) \, \frac{d\Phi \Bigl (T_\text {e}(t_\ell ), \vec {{R}}(t_\ell ) \Bigr )}{dT_\text {e}}. \end{aligned}$$

To proceed with the determination of $T_\text {e}(t_{\ell +1})$, we numerically solve the previously outlined equations relating to energy absorption and transfer. With the computed temperature $T_\text {e}(t_{\ell +1})$ and the updated position vector $\vec {R}(t_{\ell +1})$, it is then possible to ascertain various other critical parameters such as $\vec {F}(t_{\ell +1})$, $S_\text {e}(t_{\ell +1})$, and $C_\text {e}(t_{\ell +1})$. Additionally, it is beneficial to compute $G_{\text {ep}_{\mathscr {M}_k}}(t_{\ell +1})$ at this stage:

$$\begin{aligned} G_{\text {ep}_{\mathscr {M}_k}}(t_{\ell +1}) \equiv G_{\text {ep}_{\mathscr {M}_k}} \left( T_\text {e}(t_{\ell +1}), \vec {{R}}(t_{\ell +1}), \vec {{V}}(t_{\ell +1}) \right) . \end{aligned}$$

Given that $G_{\text {ep}_{\mathscr {M}_k}}$ is intricately dependent on the velocity vector $\vec {V}(t_{\ell +1})$, which remains undetermined at this stage, we adopt the velocity vector from the previous time step, $\vec {V}(t_\ell )$, as a practical approximation for computing $G_{\text {ep}_{\mathscr {M}_k}}(t_{\ell +1})$:

42 $$\begin{aligned} G_{\text {ep}_{\mathscr {M}_k}}(t_{\ell +1}) \approx G_{\text {ep}_{\mathscr {M}_k}} \left( T_\text {e}(t_{\ell +1}), \vec {{R}}(t_{\ell +1}), \vec {{V}}(t_\ell ) \right) . \end{aligned}$$

Furthermore, we get for $\vec {{V}}(t_{\ell +1})$ by inserting Eq. (32) for $\vec {{F}}_\text {tot}(t_{\ell +1})$:

$$\begin{aligned} \vec {{V}}(t_{\ell +1}) \overset{(35)}{=}&\vec {{V}}(t_\ell ) + \frac{\Delta t}{2\, m} \, \Bigl (\vec {{F}}_\text {tot}(t_\ell ) + \vec {{F}}_\text {tot}(t_{\ell +1}) \Bigr ) \nonumber \\ \overset{(32)}{=}&\vec {{V}}(t_\ell ) + \frac{\Delta t}{2\, m} \, \Bigl (\vec {{F}}_\text {tot}(t_\ell ) + \vec {{F}}(t_{\ell +1}) \Bigr ) + \frac{\Delta t}{2} \sum _{k=1}^{N_\mathscr {M}} \, \xi _{\mathscr {M}_k}(t_{\ell +1}) \, \textbf{P}_{\mathscr {M}_k} \cdot \vec {{V}}(t_{\ell +1}). \end{aligned}$$

With the definition of

43 $$\begin{aligned} \vec {{W}}(t_{\ell +1}) := \vec {{V}}(t_\ell ) + \frac{\Delta t}{2\, m} \, \Bigl (\vec {{F}}_\text {tot}(t_\ell ) + \vec {{F}}(t_{\ell +1}) \Bigr ), \end{aligned}$$

we are able to compute this quantity due to our ability to specify $\vec {F}(t_{\ell +1})$. This force vector is computed from the known position vector $\vec {R}(t_{\ell +1})$ and electron temperature $T_\text {e}(t_{\ell +1})$ by using the potential function $\Phi$. The vector $\vec {W}(t_{\ell +1})$ effectively represents the hypothetical velocity vector at time $t_{\ell +1}$, assuming electron-phonon coupling is disregarded at this step. Using the properties of the phonon mode projection operators we obtain

$$\begin{aligned}&\vec {{V}}(t_{\ell +1}) = \vec {{W}}(t_{\ell +1}) + \frac{\Delta t}{2} \sum _{k=1}^{N_\mathscr {M}} \, \xi _{\mathscr {M}_k}(t_{\ell +1}) \, \textbf{P}_{\mathscr {M}_k} \cdot \vec {{V}}(t_{\ell +1}), \hspace{50pt} \nonumber \\ \Leftrightarrow&\underbrace{\sum _{k=1}^{N_\mathscr {M}} \textbf{P}_{\mathscr {M}_k}}_{=1} \cdot \vec {{V}}(t_{\ell +1}) = \underbrace{\sum _{k=1}^{N_\mathscr {M}} \textbf{P}_{\mathscr {M}_k}}_{=1} \cdot \vec {{W}}(t_{\ell +1}) + \frac{\Delta t}{2} \sum _{k=1}^{N_\mathscr {M}} \, \xi _{\mathscr {M}_k}(t_{\ell +1}) \, \textbf{P}_{\mathscr {M}_k} \cdot \vec {{V}}(t_{\ell +1}). \end{aligned}$$

When the matrix $\textbf{P}_{\mathscr {M}_i}$ multiplies from the left, where *i* represents an arbitrary index from the set $\{1,\ldots ,N_\mathscr {M}\}$, it results in

$$\begin{aligned}&\textbf{P}_{\mathscr {M}_i} \cdot \vec {{V}}(t_{\ell +1}) = \textbf{P}_{\mathscr {M}_i} \cdot \vec {{W}}(t_{\ell +1}) + \frac{\Delta t}{2} \, \xi _{\mathscr {M}_i}(t_{\ell +1}) \, \textbf{P}_{\mathscr {M}_i} \cdot \vec {{V}}(t_{\ell +1}), \hspace{50pt} \nonumber \\ \Leftrightarrow&\textbf{P}_{\mathscr {M}_i} \cdot \vec {{W}}(t_{\ell +1}) = \left( 1 - \frac{\Delta t}{2} \, \xi _{\mathscr {M}_i}(t_{\ell +1}) \right) \textbf{P}_{\mathscr {M}_i} \cdot \vec {{V}}(t_{\ell +1}). \end{aligned}$$

Given that the index *i* was selected arbitrarily, the aforementioned equation holds for every $i \in \{1,\ldots ,N_\mathscr {M}\}$. To maintain consistency in notation, we revert *i* to *k*, concluding that for all $k \in \{1,\ldots ,N_\mathscr {M}\}$:

44 $$\begin{aligned} \textbf{P}_{\mathscr {M}_k} \cdot \vec {{V}}(t_{\ell +1}) = \frac{1}{1-\frac{\Delta t}{2} \, \xi _{\mathscr {M}_k}(t_{\ell +1})}\, \textbf{P}_{\mathscr {M}_k} \cdot \vec {{W}}(t_{\ell +1}). \end{aligned}$$

The matrix product $\textbf{P}_{\mathscr {M}_k} \cdot \vec {{V}}(t_{\ell +1})$ can be computed if the parameter $\xi _{\mathscr {M}_k}(t_{\ell +1})$ is known, given that $\vec {{W}}(t_{\ell +1})$ has already been ascertained. To determine $\xi _{\mathscr {M}_k}(t_{\ell +1})$, we examine the kinetic energy $E_{\text {kin}_{\mathscr {M}_k}}(t_{\ell +1})$ associated with the phonon mode set $\mathscr {M}_k$:

$$\begin{aligned} E_{\text {kin}_{\mathscr {M}_k}}(t_{\ell +1}) {=}&\frac{M}{2} \left( \textbf{P}_{\mathscr {M}_k} \cdot \vec {{V}}(t_{\ell +1})\right) \cdot \textbf{P}_{\mathscr {M}_k} \cdot \vec {{V}}(t_{\ell +1}) \nonumber \\ \overset{(44)}{=}&\frac{1}{\left( 1-\frac{\Delta t}{2} \, \xi _{\mathscr {M}_\ell }(t_{\ell +1})\right) ^2} \, \frac{M}{2} \left( \textbf{P}_{\mathscr {M}_k} \cdot \vec {{W}}(t_{\ell +1})\right) \cdot \textbf{P}_{\mathscr {M}_k} \cdot \vec {{W}}(t_{\ell +1}). \end{aligned}$$

We define

45 $$\begin{aligned} {H}_{\mathscr {M}_k}(t_{\ell +1}) := \frac{M}{2} \left( \textbf{P}_{\mathscr {M}_k} \cdot \vec {{W}}(t_{\ell +1})\right) \cdot \textbf{P}_{\mathscr {M}_k} \cdot \vec {{W}}(t_{\ell +1}), \end{aligned}$$

which is computable directly from the determined $\vec {{W}}(t_{\ell +1})$. The term ${H}_{\mathscr {M}_k}(t_{\ell +1})$ denotes the kinetic energy of the phonon mode set $\mathscr {M}_k$ at the time step $t_{\ell +1}$, provided the impact of electron-phonon coupling is disregarded at this specific time step. Subsequently, we obtain:

46 $$\begin{aligned} E_{\text {kin}_{\mathscr {M}_k}}(t_{\ell +1}) = \frac{{H}_{\mathscr {M}_k}(t_{\ell +1})}{1 - \Delta t \, \xi _{\mathscr {M}_\ell }(t_{\ell +1}) + \frac{\Delta t^2}{4} \, \xi _{\mathscr {M}_\ell }(t_{\ell +1})^2 } \end{aligned}$$

and obtain for the parameter $\xi _{\mathscr {M}_k}$ at time step $t_{\ell +1}$:

47 $$\begin{aligned} \xi _{\mathscr {M}_k}(t_{\ell +1}) \overset{(33)}{=}&\frac{|\mathscr {M}_k| \, G_{\text {ep}_{\mathscr {M}_k}}(t_{\ell +1}) \left( T_\text {e}(t_{\ell +1}) - T_{\text {i}_{\mathscr {M}_k}}(t_{\ell +1}) \right) }{2\, E_{\text {kin}_{\mathscr {M}_k}}(t_{\ell +1})} \nonumber \\ {=}&\frac{|\mathscr {M}_k| \, G_{\text {ep}_{\mathscr {M}_k}}(t_{\ell +1}) \left( T_\text {e}(t_{\ell +1}) - \frac{2\, E_{\text {kin}_{\mathscr {M}_k}}(t_{\ell +1})}{|\mathscr {M}_k| \, k_\text {B}} \right) }{2\, E_{\text {kin}_{\mathscr {M}_k}}(t_{\ell +1})} \nonumber \\ {=}&\frac{|\mathscr {M}_k| \, G_{\text {ep}_{\mathscr {M}_k}}(t_{\ell +1}) \, T_\text {e}(t_{\ell +1}) }{2\, E_{\text {kin}_{\mathscr {M}_k}}(t_{\ell +1})} - \frac{G_{\text {ep}_{\mathscr {M}_k}}(t_{\ell +1})}{k_\text {B}} \nonumber \\ \overset{(46)}{=}&\frac{|\mathscr {M}_k| \, G_{\text {ep}_{\mathscr {M}_k}}(t_{\ell +1}) \, T_\text {e}(t_{\ell +1}) }{2\, {H}_{\mathscr {M}_k}(t_{\ell +1})} \left( 1 - \Delta t \, \xi _{\mathscr {M}_\ell }(t_{\ell +1}) + \frac{\Delta t^2}{4} \, \xi _{\mathscr {M}_\ell }(t_{\ell +1})^2 \right) \nonumber \\&- \frac{G_{\text {ep}_{\mathscr {M}_k}}(t_{\ell +1})}{k_\text {B}}. \end{aligned}$$

Since all variables are evaluated at the time step $t_{\ell +1}$, we will omit the time argument $(t_{\ell +1})$ in subsequent expressions for conciseness. We now proceed to solve this quadratic equation for $\xi _{\mathscr {M}_k}$:

48 $$\begin{aligned}&0 = \frac{|\mathscr {M}_k| \, G_{\text {ep}_{\mathscr {M}_k}} \, T_\text {e} \, \Delta t^2 }{8\, {H}_{\mathscr {M}_k}} \, \xi _{\mathscr {M}_k}^2 - \left( 1+ \frac{|\mathscr {M}_k| \, G_{\text {ep}_{\mathscr {M}_k}} \, T_\text {e} \, \Delta t }{2\, {H}_{\mathscr {M}_k}} \right) \xi _{\mathscr {M}_k} \nonumber \\&+ \frac{|\mathscr {M}_k| \, G_{\text {ep}_{\mathscr {M}_k}} \, T_\text {e} \, }{2\, {H}_{\mathscr {M}_k}} - \frac{G_{\text {ep}_{\mathscr {M}_k}}}{k_\text {B}}, \nonumber \\ \Leftrightarrow&0 = \xi _{\mathscr {M}_k}^2 - \left( \frac{8\, {H}_{\mathscr {M}_k}}{|\mathscr {M}_k| \, G_{\text {ep}_{\mathscr {M}_k}} \, T_\text {e} \, \Delta t^2} + \frac{4}{\Delta t} \right) \xi _{\mathscr {M}_k} + \frac{4}{\Delta t^2} \nonumber \\&- \frac{8\, {H}_{\mathscr {M}_k}}{k_\text {B} \, |\mathscr {M}_k| \, G_{\text {ep}_{\mathscr {M}_k}} \, T_\text {e} \, \Delta t^2}, \nonumber \\ \Rightarrow&\xi _{\mathscr {M}_k} = \frac{4\, {H}_{\mathscr {M}_k}}{|\mathscr {M}_k| \, G_{\text {ep}_{\mathscr {M}_k}} \, T_\text {e} \, \Delta t^2} + \frac{2}{\Delta t} \nonumber \\&- \sqrt{\left( \frac{4\, {H}_{\mathscr {M}_k}}{|\mathscr {M}_k| \, G_{\text {ep}_{\mathscr {M}_k}} \, T_\text {e} \, \Delta t^2} + \frac{2}{\Delta t} \right) ^2 + \frac{8\, {H}_{\mathscr {M}_k}}{k_\text {B} \, |\mathscr {M}_k| \, G_{\text {ep}_{\mathscr {M}_k}} \, T_\text {e} \, \Delta t^2} - \frac{4}{\Delta t^2} }. \end{aligned}$$

$\xi _{\mathscr {M}_k}$ either increases or decreases the velocity of ions in alignment with the velocities of the phonon modes from the set $\mathscr {M}_k$. Given that the velocities contribute quadratically to the kinetic energy, the direction of velocity (sign) does not impact energy conservation. Consequently, there are two mathematical possibilities for $\xi _{\mathscr {M}_k}$. The first solution involves a minor adjustment in velocity, corresponding to a small value of $\xi _{\mathscr {M}_k}$. This represents the solution found in Eq. (48). Alternatively, the second solution either reverses the velocity or alters the direction of movement, correlating to a significant absolute value of $\xi _{\mathscr {M}_k}$. This outcome, deemed physically impractical, aligns with the “+” option of the quadratic equation. To confirm that the “+” solution yields a larger magnitude, consider *a* as the sum of the first two terms and *b* as the root part in Eq. (48), thus $\xi _{\mathscr {M}_k} \overset{(48)}{=}a\pm b.$ Here, *a* is non-negative by definition, and *b* is non-negative if the root generates a tangible solution. Applying the triangle inequality brings us to: $|a - b| \le |a| + |b| = a + b = |a + b|$, indicating the larger magnitude of the “+” solution, solidifying its consideration as the unphysical option.

Eq. (48) becomes inapplicable when $G_{\text {ep}_{\mathscr {M}_k}} = 0$. Under this condition, $\xi _{\mathscr {M}_k}$ is determined to be zero, following directly from its defining equation, Eq. (33). This occurs because, in the absence of electron-phonon coupling ($G_{\text {ep}_{\mathscr {M}_k}} = 0$), there is no modification induced on the ion velocities by the phonon modes from set $\mathscr {M}_k$, leading to $\xi _{\mathscr {M}_k} = 0$. Eq. (48) is also not valid for $T_\text {e}=0$. Here, we get $\xi _{\mathscr {M}_k}=- \frac{G_{\text {ep}_{\mathscr {M}_k}}}{k_\text {B}}$ from Eq. (47).

To calculate $E(t_{\ell +1})$, we define

49 $$\begin{aligned} I(t_\ell ) := \int \limits _{t_0}^{t_\ell } dt\, (S_\text {e} + C_\text {e}) \, \frac{dT_\text {e}}{dt}. \end{aligned}$$

Consequently, we derive that $I(t_0) = 0$, and $U(t_{\ell +1}) = \Phi (t_{\ell +1}) + I(t_{\ell +1})$. For the numerical computation of $I(t_{\ell +1})$, we begin with the value $I(t_\ell )$ and approximate the remaining integral from $t_\ell$ to $t_{\ell +1}$ in Eq. (49) by employing the trapezoidal rule:

50 $$\begin{aligned} I(t_{\ell + 1}) = I(t_\ell ) + \frac{1}{2} \Bigl ( \bigl (S_\text {e}(t_\ell ) + C_\text {e}(t_\ell ) \bigr ) \, \Delta T_\text {e}(t_\ell ) + \bigl (S_\text {e}(t_{\ell +1}) + C_\text {e}(t_{\ell +1}) \bigr ) \, \Delta T_\text {e}(t_{\ell +1}) \Bigr ). \ \end{aligned}$$

Now we have calculated all quantities at time step $t_{\ell +1}$ and summarize the calculation procedure:

$$\begin{aligned} \vec {{R}}(t_{\ell +1}) \overset{(34)}{=}&\vec {{R}}(t_\ell ) + \Delta t \ \vec {{V}}(t_\ell ) + \frac{\Delta t^2}{2\,m} \, \vec {{F}}_\text {tot}(t_\ell ), \nonumber \\ \Delta E_{\text {L}_\text {abs}}(t_\ell ) \overset{(36)}{=}&E_{\text {L}_\text {abs}}(t_{\ell +1}) - E_{\text {L}_\text {abs}}(t_\ell ), \nonumber \\ \Delta E_\text {ep}(t_\ell ) \overset{(37)}{=}&-\sum _{k=1}^{N_\mathscr {M}}|\mathscr {M}_k|\, G_{\text {ep}_{\mathscr {M}_k}}(t_\ell ) \left( T_\text {e}(t_\ell ) - T_{\text {i}_{\mathscr {M}_k}}(t_\ell )\right) \, \Delta t, \nonumber \\ \Delta T_\text {e}(t_\ell ) \overset{(39)}{=}&\frac{\Delta E_\text {ep}(t_\ell ) + \Delta E_{\text {L}_\text {abs}}(t_\ell )}{C_\text {e}(t_\ell )}, \nonumber \\ T_\text {e}(t_{\ell +1}) \overset{(40)}{=}&T_\text {e}(t_\ell ) + \Delta T_\text {e}(t_\ell ), \nonumber \\ S_\text {e}(t_{\ell +1}) {=}&-\frac{\partial \Phi \Bigl (T_\text {e}(t_{\ell +1}),\vec {{R}}(t_{\ell +1})\Bigr )}{\partial T_\text {e}}, \nonumber \\ C_\text {e}(t_{\ell +1}) {=}&-T_\text {e}(t_{\ell +1})\, \frac{\partial ^2 \Phi \Bigl (T_\text {e}(t_{\ell +1}),\vec {{R}}(t_{\ell +1})\Bigr )}{\partial T_\text {e}^2}, \nonumber \\ \vec {{F}}(t_{\ell +1}) \overset{(31)}{=}&\left[ \begin{array}{c} -\nabla _{\textbf{r}_1} \Phi \Bigl (T_\text {e}(t_{\ell +1}),\vec {{R}}(t_{\ell +1})\Bigr ) \\ \vdots \\ -\nabla _{\textbf{r}_{N_\text {at}}} \Phi \Bigl (T_\text {e}(t_{\ell +1}),\vec {{R}}(t_{\ell +1})\Bigr ) \end{array}\right] , \nonumber \\ G_{\text {ep}_{\mathscr {M}_k}}(t_{\ell +1}) \overset{(42)}{=}&G_{\text {ep}_{\mathscr {M}_k}} \left( T_\text {e}(t_{\ell +1}), \vec {{R}}(t_{\ell +1}), \vec {{V}}(t_\ell ) \right) , \nonumber \\ \vec {{W}}(t_{\ell +1}) \overset{(43)}{=}&\vec {{V}}(t_\ell ) + \frac{\Delta t}{2\, M} \, \Bigl (\vec {{F}}_\text {tot}(t_\ell ) + \vec {{F}}(t_{\ell +1}) \Bigr ), \nonumber \\ {H}_{\mathscr {M}_k}(t_{\ell +1}) \overset{(45)}{=}&\frac{M}{2} \left( \textbf{P}_{\mathscr {M}_k} \cdot \vec {{W}}(t_{\ell +1})\right) \cdot \textbf{P}_{\mathscr {M}_k} \cdot \vec {{W}}(t_{\ell +1}), \nonumber \\ \xi _{\mathscr {M}_k}(t_{\ell +1}) \overset{(48)}{=}&\frac{4\, {H}_{\mathscr {M}_k}(t_{\ell +1})}{|\mathscr {M}_k| \, G_{\text {ep}_{\mathscr {M}_k}}(t_{\ell +1}) \, T_\text {e}(t_{\ell +1}) \, \Delta t^2} + \frac{2}{\Delta t} - \sqrt{\begin{array}{l} \displaystyle \left( \frac{4\, {H}_{\mathscr {M}_k}(t_{\ell +1})}{|\mathscr {M}_k| \, G_{\text {ep}_{\mathscr {M}_k}}(t_{\ell +1}) \, T_\text {e}(t_{\ell +1}) \, \Delta t^2} + \frac{2}{\Delta t} \right) ^2\\[20pt] \displaystyle + \ \frac{8\, {H}_{\mathscr {M}_k}(t_{\ell +1})}{k_\text {B} \, |\mathscr {M}_k| \, G_{\text {ep}_{\mathscr {M}_k}} \, T_\text {e}(t_{\ell +1}) \, \Delta t^2} - \frac{4}{\Delta t^2} \end{array} },\nonumber \\ \vec {{V}}(t_{\ell +1}) \overset{(44)}{=}&\sum _{k=1}^{N_\mathscr {M}} \frac{1}{1-\frac{\Delta t}{2} \, \xi _{\mathscr {M}_k}(t_{\ell +1})}\, \textbf{P}_{\mathscr {M}_k} \cdot \vec {{W}}(t_{\ell +1}). \nonumber \\ \vec {{F}}_\text {tot}(t_{\ell +1}) \overset{(32)}{=}&\vec {{F}}(t_{\ell +1}) + \sum _{k=1}^{N_\mathscr {M}} \, \xi _{\mathscr {M}_k}(t_{\ell +1}) \, M \, \textbf{P}_{\mathscr {M}_k} \cdot \vec {{V}}(t_{\ell +1}), \nonumber \\ E_{\text {kin}_{\mathscr {M}_k}}(t_{\ell +1}) \overset{(32)}{=}&\frac{M}{2}\, \vec {{V}}(t_{\ell +1}) \cdot \textbf{P}_{\mathscr {M}_k} \cdot \vec {{V}}(t_{\ell +1}), \nonumber \\ T_{\text {i}_{\mathscr {M}_k}}(t_{\ell +1}) {=}&\frac{2\, E_{\text {kin}_{\mathscr {M}_k}}(t_{\ell +1})}{|\mathscr {M}_k| \, k_\text {B}}, \nonumber \\ \Delta E_{\text {L}_\text {abs}}(t_{\ell +1}) \overset{(36)}{=}&E_{\text {L}_\text {abs}}(t_{\ell +2}) - E_{\text {L}_\text {abs}}(t_{\ell +1}), \nonumber \\ \Delta E_\text {ep}(t_{\ell +1}) \overset{(37)}{=}&-\sum _{k=1}^{N_\mathscr {M}}|\mathscr {M}_k|\, G_{\text {ep}_{\mathscr {M}_k}}(t_{\ell +1}) \left( T_\text {e}(t_{\ell +1}) - T_{\text {i}_{\mathscr {M}_k}}(t_{\ell +1})\right) \, \Delta t, \nonumber \\ \Delta T_\text {e}(t_{\ell +1}) \overset{(39)}{=}&\frac{\Delta E_\text {ep}(t_{\ell +1}) + \Delta E_{\text {L}_\text {abs}}(t_{\ell +1})}{C_\text {e}(t_{\ell +1})}, \nonumber \\ I(t_{\ell +1}) \overset{(50)}{=}&I(t_\ell ) + \frac{1}{2} \Bigl ( \bigl (S_\text {e}(t_\ell ) + C_\text {e}(t_\ell ) \bigr ) \, \Delta T_\text {e}(t_\ell ) + \bigl (S_\text {e}(t_{\ell +1}) + C_\text {e}(t_{\ell +1}) \bigr ) \, \Delta T_\text {e}(t_{\ell +1}) \Bigr ), \nonumber \\ E(t_{\ell +1}) {=}&\Phi \Bigl (T_\text {e}(t_{\ell +1}),\vec {{R}}(t_{\ell +1})\Bigr ) + I(t + \Delta t). \end{aligned}$$

If this algorithm is put into practice, it becomes necessary to only retain the values of each variable at the current time step $t_{\ell +1}$ and the preceding time step $t_{\ell }$. This optimizes memory usage by eliminating the need to store data from earlier time steps beyond the most recent one, which can significantly streamline computations, especially in simulations or processes where a large number of time steps are involved.

#### Remarks

- As previously indicated, the model for electron-phonon coupling that we have developed can be incorporated within $T_\text {e}$-dependent DFT. Consequently, in this framework, $\Phi$ is equivalent to the Helmholtz free energy *F* associated with the electrons.
- This model predicts exact conservation of energy. Therefore, the described numerical implementation using the Velocity Verlet algorithm should exhibit no drift or fluctuation in the total energy expression $E+E_\text {kin} - E_{\text {L}_\text {abs}}$ as the time increment ${\Delta t}$ approaches zero. This characteristic provides a means to verify the accuracy of the algorithm’s numerical implementation within the software.
- If a set of phonon modes, denoted as $\mathscr {M}_k$, includes only a limited number of modes, the associated ionic temperature $T_{\text {i}_{\mathscr {M}_k}}$ will exhibit considerable temporal fluctuations. Specifically, if the set comprises only a single phonon mode, the corresponding ionic temperature may become ill-defined due to these fluctuations. Therefore, it is imperative that each phonon mode set $\mathscr {M}_k$ encompasses a sufficient number of modes to ensure stability and accuracy in measurements.
- When the lattice structure melts due to laser excitation, the symmetry of the structure is disrupted. Consequently, in such circumstances, applying different electron-phonon coupling constants $G_{\text {ep}_{\mathscr {M}_k}}$ for distinct phonon mode sets $\mathscr {M}_k$ may be considered non-physical. Therefore, a single coupling constant should be utilized instead to accurately reflect the altered physical conditions.
- Laser pulses with a Gaussian-shaped time profile are frequently employed in experimental settings. These pulses are characterized by their full width at half maximum (FWHM) time width, denoted as $\tau$. If $E_{\text {L}_\text {tot}}$ represents the total laser-absorbed energy of the pulse, then the rate of total laser-absorbed energy at a given time $t_\ell$ can be expressed as follows:
  51 $$\begin{aligned} \frac{dE_{\text {L}_\text {abs}}(t_\ell )}{dt} = \frac{E_{\text {L}_\text {tot}}}{\tau } \sqrt{\frac{\log (16)}{\pi }} \, \exp \left( -\frac{(t_\ell -2\,\tau )^2}{\tau ^2}\,\log (16) \right) . \end{aligned}$$
  In our molecular dynamics (MD) simulation, the initial time is set at $t_0 = 0$, and the peak energy absorption rate occurs at $t = 2\tau$. Additionally, 99.99975% of the total energy absorbed by the laser, $E_{\text {L}_\text {tot}}$, is absorbed between the times $t = 0$ and $t = 4\tau$. Employing the Gauss error function
  52 $$\begin{aligned} \text {erf}(x) = \frac{2}{\sqrt{\pi }} \int \limits _{0}^{x} dt \, \mathbb {e}^{-t^2}, \end{aligned}$$
  the total laser-absorbed energy up to time $t_\ell$ can be analytically calculated by:
  53 $$\begin{aligned} E_{\text {L}_\text {abs}}(t_\ell ) =&\int \limits _{0}^{t_\ell } dt\, \frac{dE_{\text {L}_\text {abs}}(t)}{dt} \nonumber \\ =&\frac{E_{\text {L}_\text {tot}}}{2} \Biggl ( \text {erf}\left( \sqrt{\log (65536)}\right) + \text {erf}\left( \frac{t_\ell -2\, \tau }{\tau }\, \sqrt{\log (16)} \right) \Biggr ). \end{aligned}$$
  Analogously, the total laser-absorbed energy at time step $t_\ell$ is calculated by
  54 $$\begin{aligned} \Delta E_{\text {L}_\text {abs}}(t_\ell ) =&\int \limits _{t_\ell }^{t_{\ell +1}}dt \, \frac{dE_{\text {L}_\text {abs}}(t)}{dt} \nonumber \\ =&\frac{E_{\text {L}_\text {tot}}}{2} \Biggl ( -\text {erf}\left( \frac{t_\ell -2\, \tau }{\tau }\, \sqrt{\log (16)} \right) \nonumber \\&\phantom {\frac{E_{\text {L}_\text {tot}}}{2} \Biggl (} + \text {erf}\left( \frac{t_{\ell +1}-2\, \tau }{\tau }\, \sqrt{\log (16)} \right) \Biggr ). \qquad \end{aligned}$$
- In the context of a molecular dynamics simulation utilizing a $T_\text {e}$-dependent interatomic potential $\Phi$, it is crucial that $\Phi$ demonstrates a physically specific electronic heat capacity, which is mathematically expressed as:
  $$\begin{aligned} C_\text {e} = -T_\text {e} \, \frac{\partial ^2 \Phi }{\partial T_\text {e}^2}. \end{aligned}$$
  For the simulation to yield physically meaningful results, a fundamental requirement is that the electronic specific heat $C_\text {e}$ must be non-negative, i.e., $C_\text {e} \ge 0$. This condition ensures that the simulated system adheres to the laws of thermodynamics.
- When employing a $T_\text {e}$-dependent interatomic potential $\Phi$ in MD simulations, the force exerted on any atom *i*, expressed as $-\nabla _{\textbf{r}_i} \Phi$, can be calculated solely based on the positions of neighboring atoms *j* within a defined cutoff radius $r^{(\text {c})}$ of $\Phi$. This spatial locality is advantageous for parallelizing MD simulations by subdividing the simulation cell into smaller subcells. These subcells can largely operate independently, requiring only minimal information exchange with adjacent cells. Such a parallelization strategy enables the simulation of systems comprising hundreds of millions of atoms within a reasonable timeframe. However, the feasibility of this parallelization approach may be compromised if the electron-phonon coupling is characterized by projecting onto phonon mode sets $\mathscr {M}_k$. In scenarios where electron-phonon coupling involves collective motion across all atoms of a structure in any phonon mode, the independence of subcells is undermined, as the behavior of each atom potentially influences and is influenced by distant atoms beyond its immediate neighbors in the simulation. This interdependence across the entire structure poses significant challenges to the parallel processing typically used in MD simulations. To compute any component of the total force
  $$\begin{aligned} \vec {{F}}_\text {tot} = \vec {{F}} + \sum _{k=1}^{N_\mathscr {M}} \, \xi _{\mathscr {M}_k} \, m \, \textbf{P}_{\mathscr {M}_k} \cdot \vec {{V}}, \end{aligned}$$
  it is essential to have access to the velocities of all atoms. This requirement arises because the projection component, $\textbf{P}_{\mathscr {M}_k} \cdot \vec {V}$, necessitates knowledge of the velocities across the entire atomic ensemble to accurately calculate the total force on any given atom *i*. However, this global dependence poses a significant limitation on the scalability and parallelizability of the simulation. A potential strategy to mitigate this issue involves redefining the projection operators $\textbf{P}_{\mathscr {M}_k}$ on a more localized basis. By constraining $\textbf{P}_{\mathscr {M}_k}$ to only consider the movements of atoms in the immediate neighborhood, it becomes feasible to maintain the characteristic parallel processing approach, which is crucial for accelerating large-scale molecular dynamics simulations. This local definition not only aligns with the computational framework commonly used in MD simulations but also reduces the computational overhead associated with handling global atomic interactions.

### Electronic energy and specific heat of Si

To exclusively simulate the EPC, we incorporated key thermodynamic functions, specifically the electronic specific heat $C_\text {e}(T_\text {e})$ and the electronic internal energy $E_\text {e}(T_\text {e})$, both expressed as functions of the electronic temperature $T_\text {e}$. Our focus was on a pristine diamond-like Si structure characterized by an optimal lattice parameter of $a = 0.539872$ nm. Using $T_\text {e}$-dependent DFT computations facilitated by the CHIVES 4.00^51,52^ code, we computed the Helmholtz free energy $F(T_\text {e})$ across a range of $T_\text {e}$ values. Further analysis involved fitting $F(T_\text {e})$ to an 11th-degree polynomial in $T_\text {e}$. From this polynomial representation, the electronic specific heat was derived employing the thermodynamic relationship $C_\text {e}(T_\text {e}) = -T_\text {e} \frac{\partial ^2 F(T_\text {e})}{\partial T_\text {e}^2}$. This approach allowed us to calculate $C_\text {e}(T_\text {e})$ systematically and accurately, ensuring a well-defined basis for simulating EPC effects in the context of thermal and electronic responses in Si.

55 $$\begin{aligned} C_\text {e}(T_\text {e}) = N_\text {at} \sum _{k=1}^{10} a^{(k)}_{C_\text {e}} \left( \frac{T_\text {e}}{31577\, \text {K}}\right) ^k. \end{aligned}$$

The coefficients $a^{(k)}_{C_\text {e}}$ are tabulated in TABLE 1. As we are employing a global electronic temperature $T_\text {e}$ in our simulations, the total electronic internal energy, $E_\text {e}(T_\text {e})$, can be obtained simply by

56 $$\begin{aligned} E_\text {e}(T_\text {e}) = \int \limits _0^{T_\text {e}} dT'&_\text {e} \ C_\text {e}\bigl (T'_\text {e}\bigr ). \end{aligned}$$

**Table 1:** Parametrization of the electronic specific heat $C_\text {e}(T_\text {e})$ using Eq. (55). The unit of $a^{(k)}_{C_\text {e}}$ is $\frac{\text {eV}}{\text {K atom}}$.

*k*	$a^{(k)}_{C_\text {e}}$	*k*	$a^{(k)}_{C_\text {e}}$	*k*	$a^{(k)}_{C_\text {e}}$
1	9.990955456836453E-6	2	-6.188280768791413E-4	3	0.040068462158504514
4	-0.26312331638621433	5	0.8576043019886007	6	-1.679202915977002
7	2.069435128552068	8	-1.5768394499029128	9	0.6799728970274491
10	-0.12703240946979374				

### Determination of absorbed energy for the femtosecond-laser excitation below the damage threshold

In our study, we established a simulation cell configured as $11 \times 11 \times 93$ conventional cells, incorporating a total of $N_{\text {at}} = 90024$ Si atoms. Periodic boundary conditions were implemented along the *x*- and *y*-axes, while open boundary conditions were applied along the *z*-axis to simulate a Si film with a thickness of 50 nm. To set the initial atomic coordinates and velocities, we employed the Andersen thermostat^53^, initializing the system at a temperature of $T_{\text {i}} = 300$ K.

Subsequent to setting up the simulation environment, MD simulations were conducted to emulate femtosecond-laser excitation across three distinct scenarios: excited PES and EPC, solely excited PES, and solely EPC. A temporal resolution of $\Delta t=1$ fs was utilized, simulating a Gaussian-shaped laser pulse with a full width at half maximum (FWHM) temporal width of $\tau =150$ fs, consistent with experimental configurations. To accurately determine the total energy $E_{\text {L}_\text {tot}}$ absorbed by the Si film from the experimentally measured fluence $I_{\text {L}_\text {tot}}$, the optical properties of Si require careful consideration. In the literature, Harb *et al.* utilized a femtosecond laser with a central wavelength of $\lambda = 387$ nm to excite the Si film. This wavelength corresponds to a photon energy of

57 $$\begin{aligned} E_\text {phot}= \frac{2\pi \, \hbar \, c}{\lambda } = 3.2 \, \text {eV}, \end{aligned}$$

Where $\hbar$ refers to the reduced Planck’s constant and *c* to the speed of light in vacuum. At this specific photon energy, the index of refraction of Si was identified in the literature as $n = 6.062 + 0.630i$^54^. Utilizing this complex index of refraction, we calculated the absorption coefficient of Si

58 $$\begin{aligned} \alpha _\text {abs} = \frac{4\pi }{\lambda } \, \text {Im}(n) = 0.0204569 \, \frac{1}{\text {nm}}, \end{aligned}$$

where $\text {Im}(n)$ is the imaginary part of the index of refraction *n*. In our study, we employed the ab-initio determined equilibrium atomic density, $\rho _\text {at} = 50.8414\, \text {atoms}/\text {nm}^3$, along with the experimentally measured total laser fluence, $I_{\text {L}_\text {tot}} = 5.6\, \text {mJ}/\text {cm}^2$ or equivalently $349.525\, \text {eV}/\text {nm}^2$, at the film’s surface. Using these parameters, we calculated the total energy absorbed by the laser, $E_{\text {L}_\text {tot}}$, within the 50 nm thick film.

59 $$\begin{aligned} \frac{E_{\text {L}_\text {tot}}}{N_\text {at}} = \left( 1 - \mathbb {e}^{-\alpha _\text {abs}\, d_\text {film}} \right) \frac{I_{\text {L}_\text {tot}}}{d_\text {film} \, \rho _\text {at}} \approx 0.1\, \frac{\text {eV}}{\text {atom}}, \end{aligned}$$

which we utilized in our MD simulations. We want to note that here we only take linear photon absorption into account. For higher fluences one may also include multiphoton absorption processes.

### Calculation of the Bragg peaks below damage threshold

**Fig. 5:**
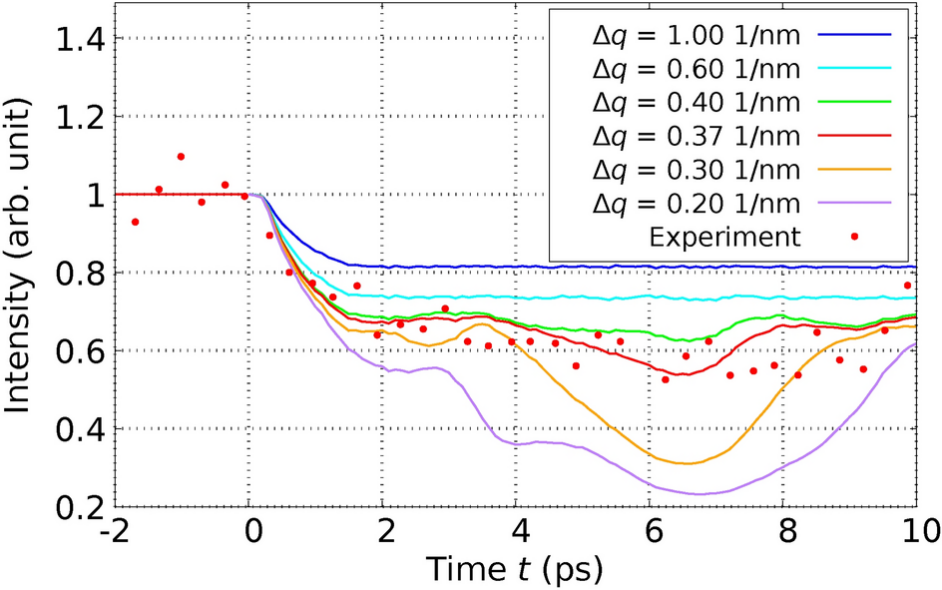
The time-dependent relative intensity of the (620) Bragg peak for various broadening parameters, $\Delta q$, is displayed. The data points shown correspond to the measured intensities of the (620) Bragg peak as reported in Fig. 4 of Ref.^28^.

**Fig. 6:**
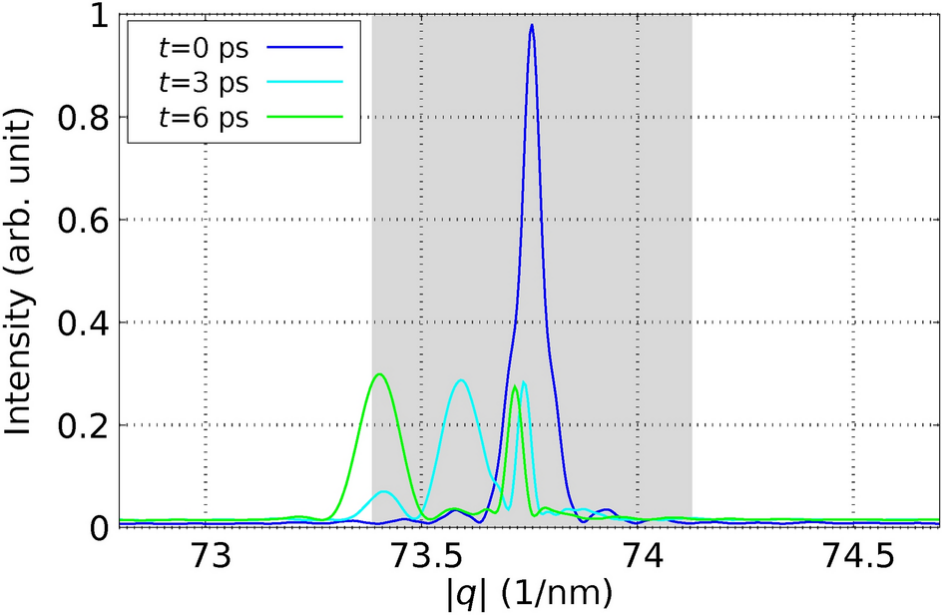
The relative scattering intensity is depicted as a function of the absolute value $|\textbf{q}|$ of the scattering vector, focusing on values proximate to $|\textbf{G}_{620}| = 73.76\, \text {nm}^{-1}$. The gray area on the graph represents the interval $\bigl [\,|\textbf{G}_{620}|-\Delta q, |\textbf{G}_{620}|+\Delta q\,\bigr ]$. This interval was chosen to facilitate a direct comparison with experimental data.

From the atomic coordinates, we derived the time-dependent intensities of the experimental studied Bragg peaks. To facilitate direct comparison with experimental results, we considered that Harb *et al.*, did not measure the intensity of individual Bragg peaks due to their use of a polycrystalline Si film. This setup generated rings, rather than spots, on the diffraction pattern, typical of monocrystalline films. Consequently, they calculated the average intensities within these rings at a specific radius and assigned this average intensity to what they designated as a Bragg peak. The assigned Bragg peak corresponds to the diffraction peak located inside the ring. To accurately derive the intensity of a measured Bragg peak (*hkl*), it was necessary to average the intensity across all Bragg peaks with scattering vector $\textbf{q}$ that meet the criterion $|\textbf{q}| \in \bigl [\,|\textbf{G}_{hkl}|-\Delta q, |\textbf{G}_{hkl}|+\Delta q\,\bigr ]$. We selected a broadening factor $\Delta q = 0.37 \, \text {nm}^{-1}$ to ensure the best possible correlation between the calculated and the experimental Bragg peak intensities. An example showing the effect of the broadening $\Delta q$ on the relative intensity of the (620) Bragg peak is presented in Fig. 5, alongside corresponding experimental data points. Several Bragg peaks possess the same absolute value $|\textbf{q}|$ of the scattering vector. The oscillations in film thickness induced by laser excitation cause shifts in $|\textbf{q}|$ for some Bragg peaks. Consequently, the $|\textbf{q}|$ of some peaks may shift outside of the intended interval $[\,|\textbf{G}_{hkl}|-\Delta q, |\textbf{G}_{hkl}|+\Delta q\,]$, significantly affecting the recorded intensities. This phenomenon is further illustrated through the (620) Bragg peak in Fig. 6, where we plot the relative intensity as a function of $|\textbf{q}|$ at selected times post-laser excitation. We also highlight the interval $[\,|\textbf{G}_{620}|-\Delta q, |\textbf{G}_{620}|+\Delta q\,]$ using a gray area.

**Fig. 7:**
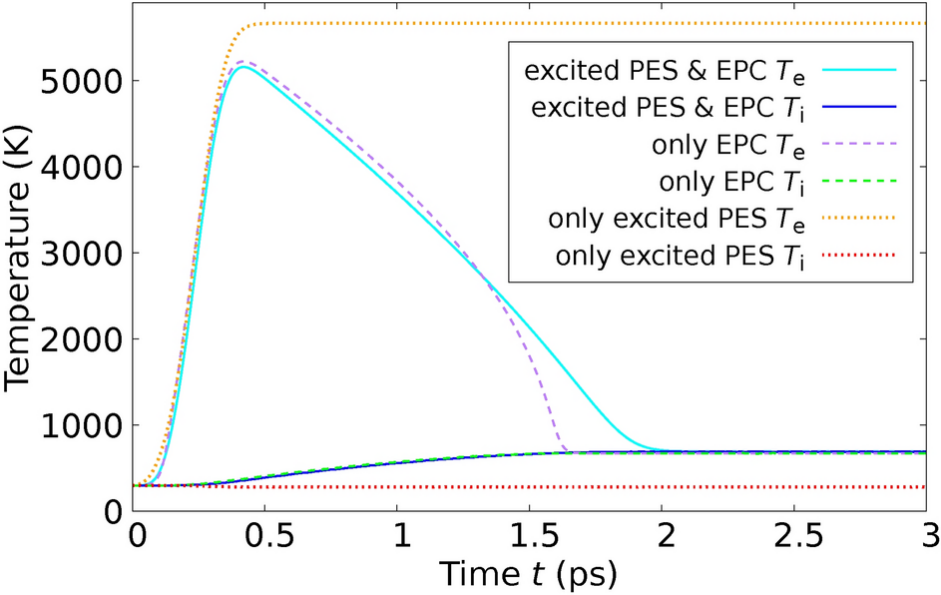
Electronic and ionic temperatures are shown as a function of time obtained from our calculations.

In Fig. 7, we present the temporal evolution of electronic $(T_\text {e})$ and ionic $(T_\text {i})$ temperatures derived from our three distinct MD simulation scenarios. Initially, the electronic temperature $T_\text {e}$ increases as a result of laser excitation. Subsequently, $T_\text {e}$ decreases while $T_\text {i}$ rises due to EPC, continuing until both temperatures equilibrate at the same value.

### Simulations for femtosecond-laser excitation above the damage threshold

We constructed a simulation cell comprising $11 \times 11 \times 56$ conventional units, containing a total of $N_\text {at}=54208$ Si atoms to simulate a 30 nm thick Si film. The periodic boundary conditions were imposed in the *x*- and *y*-directions, while open boundary conditions were applied in the *z*-direction (the $[\overline{1}11]$ direction of the crystal lattice). To establish initial atomic coordinates and velocities corresponding to a temperature of $T_\text {i}=300$ K, we utilized the Andersen thermostat^53^. The MD simulations of the femtosecond-laser excitation were subsequently carried out under three different scenarios: excited PES and EPC, excited PES alone, and EPC alone. The simulations were performed with a time step of $\Delta t = 1$ fs. We modeled the laser excitation using a Gaussian-shaped pulse with a FWHM temporal width of $\tau = 150$ fs, mirroring the experimental conditions. The total energy, $E_{\text {L}_\text {tot}}$, absorbed by the 30 nm thick film from the laser was also specified in accordance with experimental values:

60 $$\begin{aligned} \frac{E_{\text {L}_\text {tot}}}{N_\text {at}} = \left( 1 - \mathbb {e}^{-\alpha _\text {abs}\, d_\text {film}} \right) \frac{I_{\text {L}_\text {tot}}}{d_\text {film} \, \rho _\text {at}} \approx 1.2\, \frac{\text {eV}}{\text {atom}}. \end{aligned}$$

The total energy absorbed by the 30 nm thick Si film from the laser, $E_{\text {L}_\text {tot}}$, was calculated using the absorption coefficient $\alpha _\text {abs} = 0.0204569 \, \text {nm}^{-1}$ at a wavelength of 387 nm as used in the experiment^29^. This value was combined with the ab-initio obtained equilibrium atomic density $\rho _\text {at} = 50.8414 \, \text {atoms/nm}^3$, and the experimental absorbed laser fluence $I_{\text {L}_\text {tot}} = 65 \, \text {mJ/cm}^2$ equivalent to $4056.98 \, \text {eV/nm}^2$ at the surface. This combination of parameters enabled an accurate estimation of the energy absorption dynamics in the simulated Si film.

### Calculation of the Bragg peak above the damage threshold

**Fig. 8:**
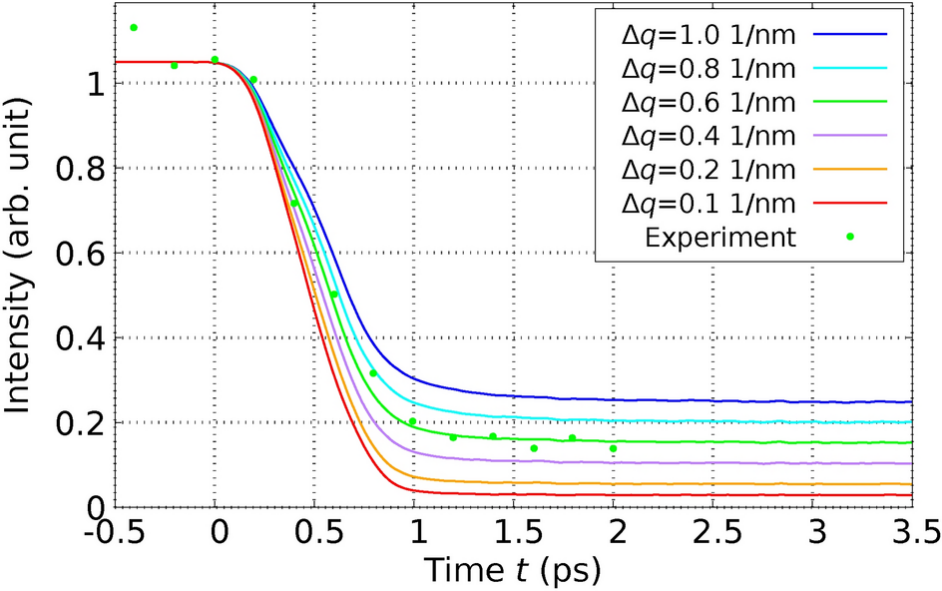
The time-dependent relative intensity of the (220) Bragg peak is depicted for several values of the broadening parameter, $\Delta q$. The data points corresponding to the measured intensities of the (220) Bragg peak are extracted from Fig. 3(c) of Ref.^29^.

The time-dependent intensity of the (220) Bragg peak was inferred from the atomic coordinates by considering all Bragg peaks within the interval $\bigl [\,|\textbf{G}_{220}|-\Delta q, |\textbf{G}_{220}|+\Delta q\,\bigr ]$. This approach was adopted to align with the methodology used by Harb *et al.*, who averaged intensities within a ring on the measured diffraction image to determine the (220) Bragg peak intensities. In Fig. 8, we display the calculated time-dependent intensities for different values of $\Delta q$ from the MD simulations that integrated both the excited PES and EPC, alongside the results published by Harb. The choice of $\Delta q$ predominantly influences the residual intensity observed after the decay of the initial Bragg peak. An increase in $\Delta q$ corresponds to a higher measured background intensity, due to the inclusion of more diffuse scattering within the evaluated range. A value of $\Delta q=0.6\, \text {nm}^{-1}$ was selected as it best replicated the residual intensity observed in the experimental data according to our calculations.

The simulations vividly demonstrate that when EPC is incorporated, there is a noticeable elevation in the final ionic temperature. This increase becomes even more pronounced when the excited PES is included alongside EPC. The presence of an excited PES typically results in the weakening of atomic bonds, which, in turn, allows for more significant energy transfer from electrons to ions, thereby elevating $T_\text {i}$. This outcome highlights the critical that both electron-phonon interactions and the state of the potential energy surface play in determining the thermal response of materials subjected to intense laser excitation.

**Fig. 9:**
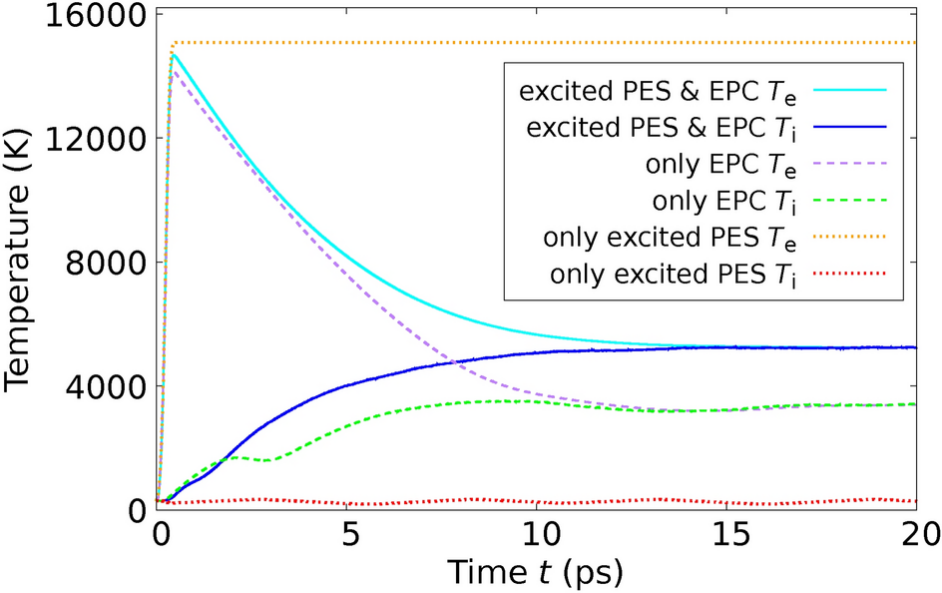
The electronic and ionic temperatures of the 30 nm Si film, as a function of time, are depicted based on our computational findings.

### Analyse of the numerical energy drift during the MD simulations

**Fig. 10:**
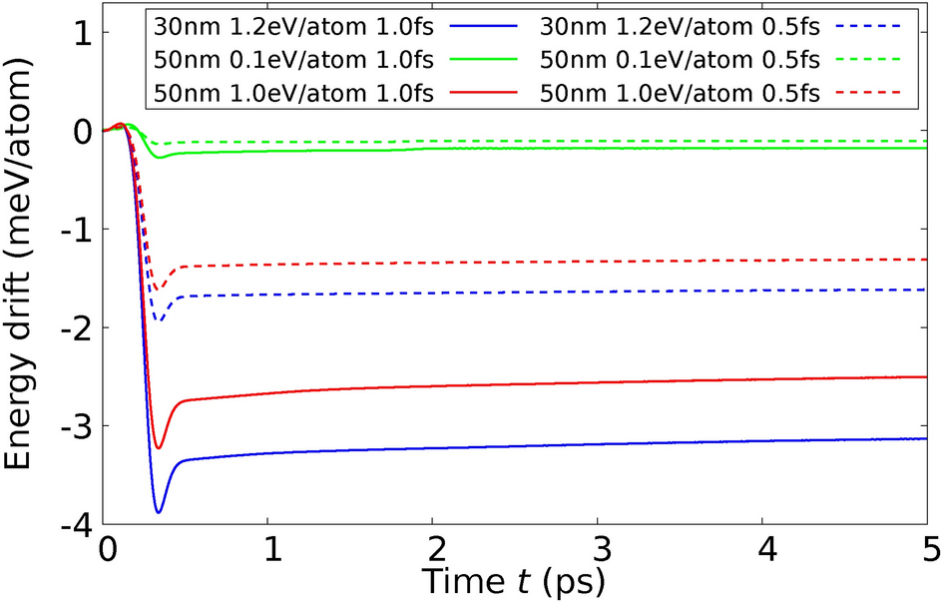
Numerical energy drift of our MD simulations for the three presented calculations to compare with the Bragg peaks with a timestep of 1 fs (solid lines) and 0.5 fs (dashed lines).

In order to analyze the stability of our implementation of the Velocity Verlet algorithm, we considered the numerical energy drift of our MD simulations:

61 $$\begin{aligned} E_\text {drift}(t) = E(t) - E_{\text {L}_\text {abs}}(t) - E(t=0), \end{aligned}$$

where *E*(*t*) denotes the energy expression Eq. (12) in the main manuscript and $E_{\text {L}_\text {abs}}(t)$ (see Eq. (53)) the total energy absorbed from the laser field at time *t*. In Fig. 10 we present the numerical energy drift as a function of time for the three MD simulations, which we present here in order to compare with the experimental measured Bragg peaks. We used a time step of 1 fs and a time step of 0.5 fs. During the action of the laser pulse, there is a small negative drift of the energy, whereas after the laser pulse there is almost no drift. The reduction of the time step decreases significantly the energy drift, especially at higher laser fluences, which shows that our implementation is correct. In addtion, the resulting Bragg peak decay is identical for both time steps.

## Data Availability

The datasets used and analysed during the current study available from the corresponding author on reasonable request.
